# Evaluation of the Pharmaceutical Activities of Chuanxiong, a Key Medicinal Material in Traditional Chinese Medicine

**DOI:** 10.3390/ph17091157

**Published:** 2024-08-31

**Authors:** Shiwei Huang, Jiamei Chen, Xiaohua Liu, Chunxin Xing, Lu Zhao, Kelvin Chan, Guanghua Lu

**Affiliations:** 1 State Key Laboratory of Southwestern Chinese Medicine Resources, School of Pharmacy, Chengdu University of Traditional Chinese Medicine, Chengdu 611137, China; huangshiwei@stu.cdutcm.edu.cn (S.H.); chenjiamei@stu.cdutcm.edu.cn (J.C.); liuxiaohua@stu.cdutcm.edu.cn (X.L.); xingchunxin@stu.cdutcm.edu.cn (C.X.); 2 Research Institute of Chinese Medicines as Drug & Food, Chengdu University of Traditional Chinese Medicine, Chengdu 611137, China; 3 Sichuan Institute for Drug Control (Sichuan Testing Center of Medical Devices), Chengdu 611731, China; zhaol@scsyjs.org; 4 Centre for Natural Products Discovery, School of Pharmacy & Biomolecular Sciences, Liverpool John Moores University, Liverpool L3 3AF, UK; 5 NICM Health Research Institute, Western Sydney University, Sydney, NSW 1797, Australia

**Keywords:** *Ligusticum chuanxiong*, Szechwan lovage rhizome, blood stasis syndrome, cardiocerebrovascular disorder, bioassay, pharmaceutical activity

## Abstract

Szechwan lovage rhizome (**SLR**, the rhizome of *Ligusticum chuanxiong* Hort., *Chuanxiong* in Chinese transliteration) is one Chinese materia medica (CMM) commonly used to activate blood circulation and remove blood stasis. SLR is applicable to most blood stasis syndromes. It has significant clinical efficacy in relation to human diseases of the cardiocerebrovascular system, nervous system, respiratory system, digestive system, urinary system, etc. Apart from China, SLR is also used in Singapore, Malaysia, the European Union, and the United States of America. However, the current chemical markers in pharmacopeia or monography for the quality assessment of SLR are not well characterized or specifically characterized, nor do they fully reflect the medicinal efficacy of SLR, resulting in the quality of SLR not being effectively controlled. CMM can only have medicinal efficacy when they are applied in vivo to an organism. The intensity of their pharmaceutical activities can more directly represent the quality of CMM. Therefore, the chemical constituents and pharmacological actions of SLR are reviewed in this paper. In order to demonstrate the medicinal efficacy of SLR in promoting blood circulation and removing blood stasis, bioassay methods are put forward to evaluate the pharmaceutical activities of SLR to improve hemorheology, hemodynamics, and vascular microcirculation, as well as its anti-platelet aggregation and anticoagulation properties. Through comprehensive analyses of these pharmaceutical properties, the quality and therapeutic value of SLR are ascertained.

## 1. Introduction

Szechwan lovage rhizome (**SLR**, *Chuanxiong* in Chinese transliteration) is derived from the dried rhizome of *Ligusticum chuanxiong* Hort. (family: Apiaceae) and has a long history of medicinal uses [1]. The earliest name for SLR was “Xiongqiong”, recorded in *The Classic of Mountains and Seas* compiled in China during the Pre-Qin Dynasty (estimated from 476 BC to 221 BC), which stated that “the grasses were many peonies and Xiongqiong”. In *Shennong’s Herbal Classic*, compiled during the Eastern Han Dynasty (more than 2000 years ago), SLR was recorded under the name of “Xiongqiong”, which was classified into the top-grade herb category [2]. Later, with the accumulation of clinical experience, ancient medical practitioners discovered that the therapeutic efficacy of SLR cultivated in Sichuan Province in China was better than that of SLR cultivated in other areas; Sichuan therefore became a genuine cultivating area for SLR. Replacement of the name “Xiongqiong” with “Chuanxiong” was first recorded in the Chinese medical classic *Prescriptions of the Bureau of Taiping People’s Welfare Pharmacy* [3]. Currently, the quantity of the production of SLR in Sichuan Province is accepted as indicative of it being a good agricultural area, amounting to more than 95% of the SLR produced in China [4].

SLR is commonly used to activate blood circulation and remove blood stasis, with efficacy in activating blood circulation and promoting *qi*, dispelling wind, and relieving pain in the practice of Chinese medicine. In particular, SLR is used as a major constituent of the composite formulae (*Fufang* in Chinese) used to cure diseases of the cardiocerebrovascular system, e.g., by offering anti-cerebral ischemia, anti-inflammatory, anti-tumor, and anti-oxidation properties, as well as protection against nerve-related symptoms, etc. [5,6,7,8,9]. According to the World Health Organization’s report, cardiovascular diseases are the leading cause of human death. The number of people dying annually from cardiovascular diseases is greater than that from any other cause of death [10]. SLR composite formulae have significant therapeutic effects on cardiocerebrovascular diseases [11,12], as well as good ameliorative and protective effects on the nervous and respiratory systems [13,14]. Meanwhile, as a health food, SLR has the effect of enhancing immunity, improving sleep, assisting in regulating blood lipids, and acting as a beautifying aid [15]. At present, besides being a decoction piece in prescription formulae, 246 patented Chinese medicines (over-the-counter (OTC) products) that contain SLR are recorded in the 2020 edition of *Chinese Pharmacopoeia*, accounting for 15.3% of the number of OTC Chinese medicines in this pharmacopoeia. Meanwhile, SLR has been used as a raw material in healthcare products. There are 82 healthcare products that contain SLR on the market in China [4]. SLR is also consumed as food by folk people. Its stems and leaves are made into salads, stir-fried into teas, and brewed into wine. The essential oil of SLR is one ingredient used [16]. In addition, about 2000 tons/year of SLR are exported from China to Singapore, Malaysia, the European Union, etc. [17]. An international standard for SLR was announced by the International Organization for Standardization in 2024 [18].

The study of the chemical constituents of SLR started in the 1950s. Up to now, more than 200 chemical compounds have been separated and identified in SLR, including phthalides, organic acids and their esters, alkaloids, polysaccharides, and other types of constituents. A large number of pharmacological studies on SLR and its chemical compounds have been carried out [19]. However, only the contents of the ethanol-soluble extract and ferulic acid were chosen as chemical markers to assess SLR’s quality and efficacy in the 2020 edition of *Chinese Pharmacopoeia*. Although the pharmacological effects of ferulic acid partly resemble those of SLR, ferulic acid is widely present throughout plantae and is not a specific compound in SLR. Therefore, the specificity and effectiveness are not sufficient in assessing the quality of SLR using the ethanol-soluble extract and ferulic acid [20]. In addition, although the pharmacological effects of the phthalides in the volatile oil of SLR are significant, these single components are unstable and easily degraded, resulting in them being unsuitable for use as chemical markers to assess the quality of SLR. The alkaline compound ligustrazine (tetramethylpyrazine) was once mistakenly regarded as a characteristic compound in SLR owing to its significant medicinal efficacy. Nevertheless, the content of ligustrazine in SLR is extremely low and cannot be found in most SLR samples [21,22]. So, ligustrazine cannot be chosen as a chemical marker to assess the quality of SLR. Hence, it is necessary to identify a novel method to effectively assess the quality of SLR.

Chinese materia medica (CMM) are the substances used to cure and prevent diseases, and they only play a role in organisms. The intensity of their pharmaceutical activity can more directly represent the quality of CMM. For example, a method for evaluating the quality of leeches according to their anticoagulant effect and the guidelines for bioassays were recorded in the *Chinese Pharmacopoeia* (2020 edition) [1]. Therefore, the chemical constituents and pharmacological effects of SLR are reviewed in this paper. Reasons for applying pharmacological effects related to SLR’s therapeutic efficacy in activating blood circulation and removing blood stasis, and its bioassay methods, are further proposed in order to effectively evaluate the pharmaceutical activities and quality of SLR.

## 2. Materials and Methods

A comprehensive review was conducted by searching the PubMed, SciFinder, and CNKI databases to check for articles related to the chemical constituents of SLR and its action on blood circulation and blood stasis from 2010 to the present. The keywords used included “Ligusticum chuanxiong”, “Ligusticum chuanxiong/activating blood circulation and removing blood stasis”, “Ligusticum chuanxiong/hemorheology”, “Ligusticum chuanxiong/hemodynamics”, “Ligusticum chuanxiong/vascular micro-circulation”, “Ligusticum chuanxiong/antiplatelet aggregation”, and “Ligusticum chuanxiong/anticoagulation”.

**Figure 1:**
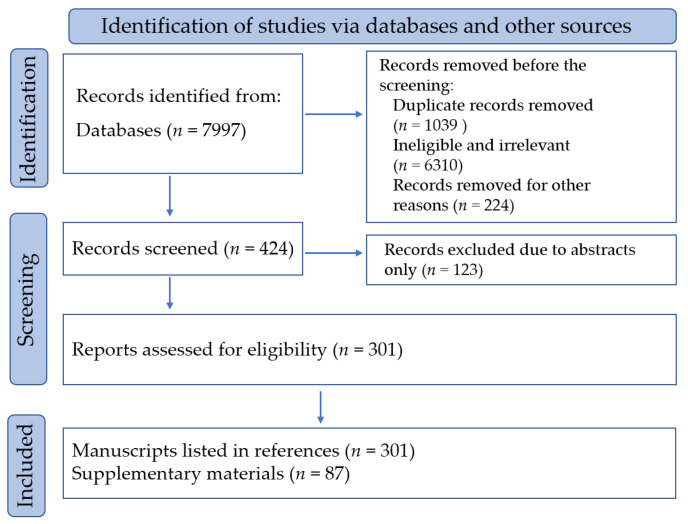
Flow chart of reference selection.

The initial search found 7997 articles from the sources, such as research articles, clinical trial databases, and relevant industry publications. After screening for title relevance and removing duplicates, 7693 articles were excluded. The included literature was manually checked to ensure that relevant information was included. The inclusion criteria were original research articles or reviews focused on the isolation of chemical constituents of SLR and the activation of blood circulation and the removal of blood stasis by SLR. The exclusion criteria included conference abstracts, editorials, non-peer-reviewed articles, duplicate articles, etc. (Figure 1).

## 3. Chemical Constituents

In 1957, Ke et al. studied the chemical constituents of SLR collected from Guanxian County, Dujiangyan City, Sichuan Province, in China [23]. They obtained alkaloids, organic acids, phenolic compounds, and neutral compounds. Owing to the limitations of scientific technology at that time, only ferulic acid was identified. At present, 276 chemical compounds have been isolated and identified in SLR, including phthalides, organic phenolic acids and their esters, alkaloids, polysaccharides, terpenes, and other types of constituents (Figure S1).

### 3.1. Phthalides

Phthalides are a class of natural products with a 5/6 bicyclic skeleton consisting of γ-lactone and a six-membered ring, in which the C-3 is mostly replaced by n-butyl or n-butenyl groups [24]. These compounds are mainly found in the plants of the Apiaceae family, such as SLR, Chinese Angelica (*Danggui*), and Chinese lovage (*Gaoben*) [25]. Phthalides are the most abundant and diverse constituents in SLR and are also the main constituents in SLR volatile oil [26]. There are 146 phthalide compounds that have been identified in SLR. On the basis of the chemical structure of these compounds, phthalide compounds are classified into monomer phthalides and dimer phthalides.

#### 3.1.1. Monomeric Phthalides

**Figure 2:**
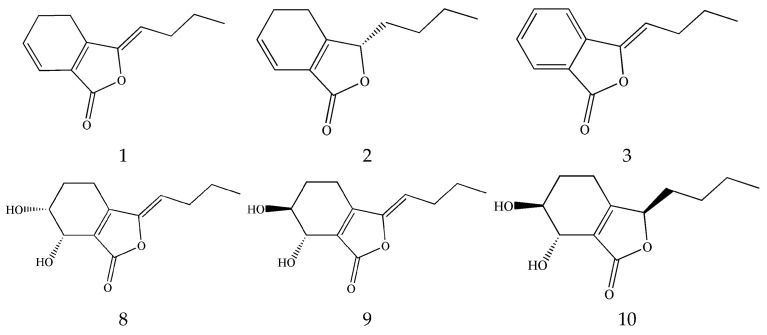
Chemical structures of main monomeric phthalide components in Szechwan lovage rhizome. 1. *Z*-ligustilide, 2. Senkyunolide A, 3. 3-butylidenephthalide, 8. Senkyunolide H, 9. Senkyunolide I, 10. Senkyunolide J.

A total of 82 monomeric phthalides and their derivatives have been isolated and identified in SLR, which are classified into alkyl and hydroxy phthalide compounds according to the type of substituent group. The main components of alkyl phthalides are *Z*-ligustilide, senkyunolide A, 3-butylidenephthalide, etc. The main components of hydroxy phthalides are senkyunolide H, senkyunolide I, and senkyunolide J (Figure 2). Among these, the content of *Z*-ligustilide reaches 1% in SLR and 58% in the volatile oil of SLR [27]. However, these major phthalide components are oils or liquids with unstable chemical properties, resulting in them being easily oxidized or decomposed by light. Li et al. showed that the purity of *Z*-ligustilide decreased from 99.48% to 41.97% after storage for 15 days at ambient temperature and decreased from 99.48% to 86.67% after 15 days of storage at a low temperature of 4 °C. However, *Z*-ligustilide was generally stable when stored at −20 °C [28]. Zuo et al. analyzed the degradation compounds of *Z*-ligustilide, senkyunolide A, and senkyunolide I using UPLC-QTOF-MS and NMR. They found that the degradation pathways of Z-ligustilide through oxidation, hydrolysis, and isomerization produced senkyunolide I, senkyunolide H, (*E*)-6, 7-trans-dihydroxyligustilide, (*Z*)-6, 7-epoxyligustilide, and (*E*)-6, 7-epoxyligustilide. They also found that oxygen was the main factor facilitating the changes in senkyunolide A and senkyunolide I. Senkyunolide A is completely converted into butylphthalide by dehydrogenation. Senkyunolide I is partially converted into its isomer (*E*)-6, 7-trans-dihydroxyligustilide by isomerization [29]. Furthermore, Duric et al. found that the mass balance and stability of ligustilide helped to bridge the gap between plant instability and bioactivity. It is worth noting that *Z*-ligustilide, senkyunolide A, and senkyunolide I are liquid and unstable at an ambient temperature [30].

#### 3.1.2. Dimeric Phthalides

**Figure 3:**
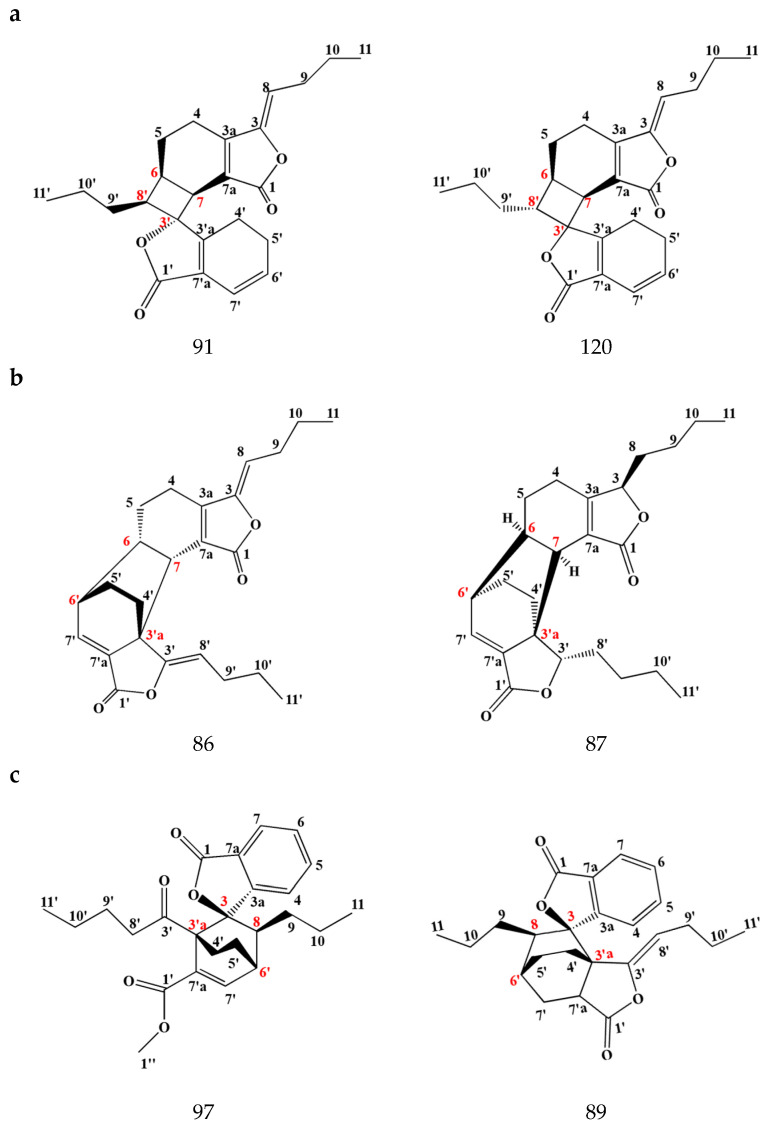

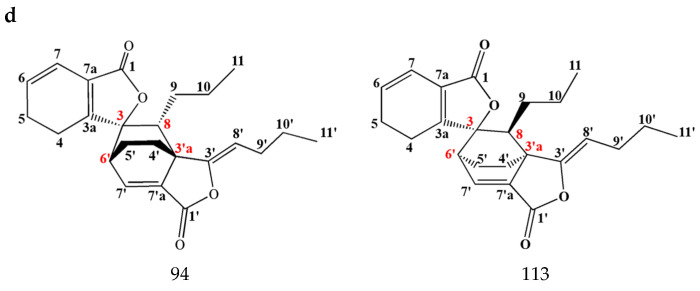
Chemical structures of various linkage types of dimeric phthalides in Szechwan lovage rhizome. (**a**) C-6,8′,7,3′-type: riligustilide (**91**), chaxiongnolide G (**120**). (**b**) C-6,6′,7,3′a-type: levistolide A (**86**), 3,8-dihydro-diligustilide (**87**). (**c**) C-3,3′a,8,6′-type: wallichilide (**97**), ansaspirolide (**89**). (**d**) C-3,6′,8′, 3′a-type: *Z*-ligustilide dimer E-232 (**94**), (3Z)-(3aR,6S,3′R,8S)-3a,8′,6,3′-diligustilide (**113**).

Dimeric phthalides are produced by the polymerization of two molecules of monomer phthalide components through chemical reactions such as the Diels–Alder reaction, cycloaddition, etc. In general, these pure compounds are in a crystalline state [25]. Wang et al. isolated and identified the first dimeric phthalide from SLR, named wallichilide, in 1985 [31]. Up to now, more than 60 dimeric phthalides have been isolated and identified in SLR. The linkage sites of monomer phthalides are different in dimeric phthalides. The common linkage sites are summarized into four types. The first type is C-6,8′,7,3′, with these compounds including riligustilide, chaxiongnolide G, *Z*-6,8′,7,3′-diligustilide, angelicide, gelispirolide, etc. The second type is C-6,6′,7,3′a, including compounds such as levistolide A, senkyunolide P, senkyunolide O, 3,8-dihydro-diligustilide, demethywallichilide, etc. The third type is C-3,3′a,8,6′, e.g., wallichilide, ansaspirolide, chuanxiongdiolide R1, methyl ester derived from angeolide, etc. The fourth type is C-3,6′,8′,3′a, including *Z*-ligustilide dimer E-232, (3Z)-(3aR,6S,3′R,8S)-3a,8′,6,3′-diligustilide, chuanxiongdiolide R7, and so on (Figure 3). Owing to the fact that the identification of the chiral and enantiomeric structures of dimers is the focus but also difficult in chemical structures, the absolute configuration of some of the dimeric phthalides reported in SLR should be elucidated further [32,33]. Most of the dimeric phthalides found in SLR are composed of monomeric phthalides, such as ligustilide, senkyunolide A, and N-butylidenephthalide. These dimeric phthalides have more stable chemical properties and significant pharmacological effects, thus being prospective chemical markers for assessing the quality of SLR.

### 3.2. Organic Phenolic Acids and Their Esters

**Figure 4:**
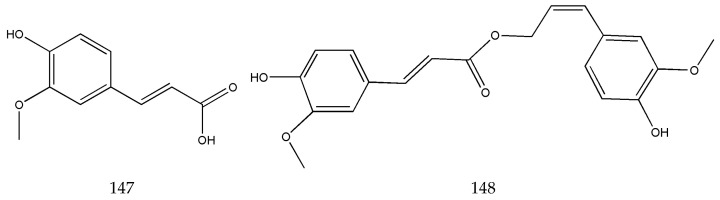

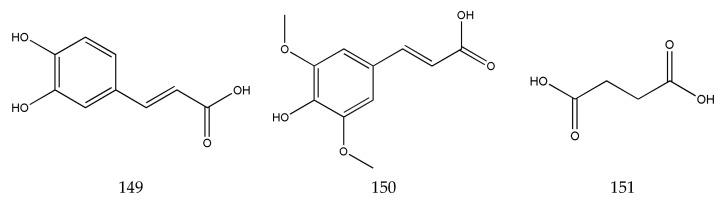
Major organic phenolic acids and their esters in Szechwan lovage rhizome. Ferulic acid (**147**), coniferyl ferulate (**148**), caffeic acid (**149**), sinapic acid (**150**), and succinic acid (**151**).

Organic acids and their esters are abundant in SLR. At present, a total of 55 organic phenolic acids and their ester components have been identified in SLR [31,34,35,36], in which the main components are ferulic acid (**147**), coniferyl ferulate (**148**), caffeic acid (**149**), sinapic acid (**150**), succinic acid (**151**), etc. (Figure 4). Ferulic acid and coniferyl ferulate are the most abundant compounds in these chemical types. Ferulic acid mainly exists in the form of coniferyl ferulate in SLR. During the drying process of medicinal materials, coniferyl ferulate is hydrolyzed to produce ferulic acid until equilibrium is reached [37]. SLR decoction is its most common application in Chinese medicine. SLR decoction pieces are boiled in water. Coniferyl ferulate can be completely hydrolyzed into ferulic acid during high-temperature boiling [38]. Ferulic acid and its sodium salt have anti-platelet aggregation and anticoagulation effects. It is related to the therapeutic efficacy of SLR in activating blood circulation and removing blood stasis [39,40]. The 2020 edition of *Chinese Pharmacopoeia* stipulates that the content of ferulic acid should be no less than 0.1% as the standard for quality control of SLR [1]. Due to the widespread distribution of ferulic acid in the plant kingdom, ferulic acid is not specific to SLR. Ferulic acid cannot fully reflect SLR’s pharmaceutical activities, resulting in it being hard to effectively assess SLR’s quality. Meanwhile, Mei et al. investigated the correlation between quality and the bioactive compounds in SLR and the diversity of their indicators. They concluded that it was not reasonable to only use ferulic acid as an indicator to control SLR’s quality. By analyzing the correlation between its biological activity in terms of anti-platelet aggregation and its chemical components, they considered *Z*-ligustilide, coniferyl ferulate, 3,5-O-dicaffeoylquinic acid, cryptochlorogenic acid, butylidenephthalide, and neocnidilide as chemical components that could be used to assess SLR’s quality [41].

### 3.3. Alkaloids

**Figure 5:**
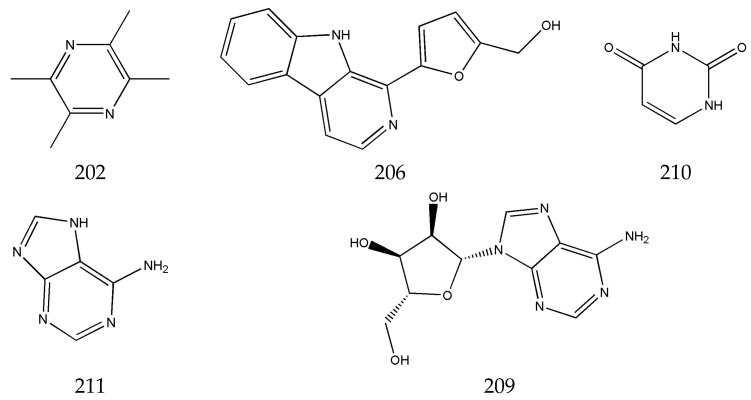
Major alkaloidal compounds in Szechwan lovage rhizome. Tetramethylpyrazine (**202**), perlolyrine (**206**), adenosine (**209**), uracil (**210**), adenine (**211**).

The alkaloids in SLR include 21 compounds, such as tetramethylpyrazine (**202**), perlolyrine (**206**), adenosine (**209**), uracil (**210**), adenine (**211**), etc. (Figure 5). The content of such components in SLR is extremely low, and they are difficult to obtain as pure compounds. The first report of obtaining tetramethylpyrazine from SLR was in 1977, by the Beijing Institute of the Pharmacy Industry [42]. In subsequent articles on the chemical constituents of SLR, pure tetramethylpyrazine has hardly ever been obtained [43,44]. HPLC analyses have shown that the tetramethylpyrazine content was about 2 μg/g in SLR, and tetramethylpyrazine was not found in some SLR samples [21]. Tetramethylpyrazine shows strong pharmacological effects with similarity to those of SLR, i.e., antithrombotic, anti-ischemic reperfusion injury, anti-tumor, and protective effects on the brain, nerves, heart, blood vessels, lungs, liver, and kidneys [45,46,47,48,49]. So, in the early literature, tetramethylpyrazine was often considered a pharmacodynamic component in SLR and a chemical marker for quality assessment of SLR [50,51,52]. In fact, tetramethylpyrazine is obtained by synthesis in pharmacological studies and in production in the pharmaceutical industry [53,54]. Nowadays, tetramethylpyrazine is not used as chemical marker for the quality assessment of SLR in the newly published literature [55,56].

### 3.4. Polysaccharides

**Figure 6:**
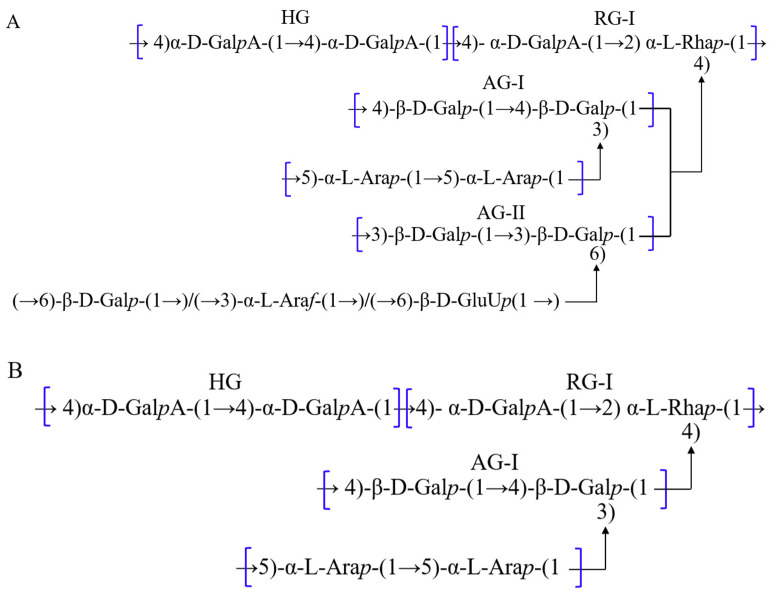
Linkage type of polysaccharides in Szechwan lovage rhizome. (**A**) LCP-I-I and (**B**) LCP-II-I. (HG) homo-galacturonan; (RG-I) rhamnogalacturonan type I; (AG-I) arabinogalactan type I; (AG-II) arabinogalactan type II.

Polysaccharides, also referred to as polyglucans, are polymers formed through the linkage of multiple monosaccharide groups by glycosidic bonds. When polysaccharides are completely hydrolyzed, the glycosidic bond is cleaved to form monosaccharides. Polysaccharides are widely found in plants, algae, fungi, etc. [57]. Fan et al. first extracted polysaccharides from SLR. Four homogeneous polysaccharide fractions were obtained by DEAE fiber column chromatography classification [58]. In recent years, SLR polysaccharides have been studied more extensively and intensively. Nine polysaccharides with different structural compositions have been obtained and characterized from SLR using aqueous, enzyme, ultrasonic, and microwave extraction methods [59]. These polysaccharides are classified into acidic pectic polysaccharides and neutral polysaccharides. Acidic pectin polysaccharides, e.g., LCP-I-I, LCP-II-I, LCXP-3a, and LCX 2, are mainly composed of galacturonic acid, galactose, arabinose, and rhamnose, which are generally linked by α-1,4-D-GalpA, α-1,2-L-Rhap, α-1,5-L-Araf, β-1,3-D-Galp, and β-1,4-D-Galp (Figure 6). LCP-I-I and LCP-II-I are typical acidic pectic polysaccharides with homo-galacturonan (HG) and rhamnogalacturonan type I (RG-I) regions as their main chains. Arabinogalactan type I and type II (AG-I/AG-II) are seen as side chains in LCP-I-I. But LCP-II-I only has AG-I as a side chain. These types of acidic pectic polysaccharides have antioxidant, immune, anti-tumor, and antibacterial activities [60,61,62].

### 3.5. Terpenes

**Figure 7:**
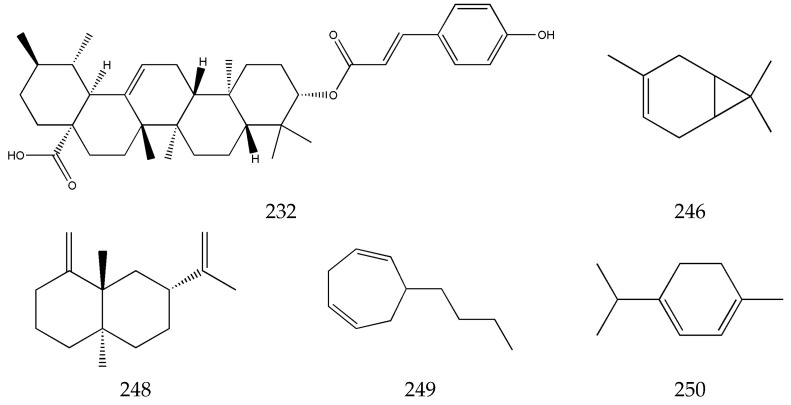
Major terpenoids in Szechwan lovage rhizome. Xiongterpene (**232**); 3-carene (**246**); eudesma-4,11-dlene (**248**); 6-butyl-1,4-cycloheptadiene (**249**); terpinene (**250**).

The terpenes in SLR are present in its volatile oil, mainly including xiongterpene (**232**), 3-carene (**246**), eudesma-4,11-dlene (**248**), 6-butyl-1,4-cycloheptadiene (**249**), terpinene (**250**), etc. [63,64,65]. There are not any reports on studies of the pharmacological effects on these compounds (Figure 7).

### 3.6. Others

**Figure 8:**
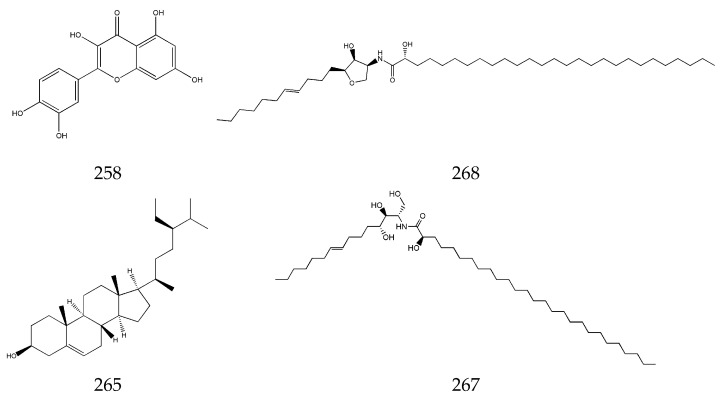
Other types of constituents in Szechwan lovage rhizome. Apigenin (**258**), β-sitosterol (**265**), (2R)-2-hydroxy-N [(2S, 3S, 4R, 8E)-1,3, 4-trihydroxypentadec-8-en-2-yl] heptacosanamide (**267**), (2R)-2-hydroxy-N-{(3S, 4S, 5S)-4-hydroxy-5-[(4E)-undec-4-en-1-yl] tetrahydrofuran-3-yl} heptacosanamide (**268**).

Small amounts of flavonoids, steroids, ceramides, and cerebrosides have been isolated from SLR, such as apigenin (**258**), quercetin (**259**), β-sitosterol (**265**), (2R)-2-hydroxy-N [(2S, 3S, 4R, 8E)-1,3, 4-trihydroxypentadec-8-en-2-yl] heptacosanamide (**267**), (2R)-2-hydroxy-N-{(3S, 4S, 5S)-4-hydroxy-5-[(4E)-undec-4-en-1-yl] tetrahydrofuran-3-yl} heptacosanamide (**268**), (2R) 2-hydroxy-N-[(2S, 3S, 4R, 8E)-1,3, 4-trihydroxyicos-8-en-2-yl] tetracosanamide (**269**), and other components [66,67] (Figure 8).

## 4. Pharmaceutical Activities

SLR is a key type of CMM in Chinese medicine used to activate blood circulation and remove blood stasis. Many clinical trials and pharmacological studies on SLR extracts, single compounds, and Chinese medicine formulae have shown its significant efficacy for diseases of the cardiocerebrovascular system, the respiratory system, the nervous system, the digestive system, and the urinary system (Table 1).

### 4.1. The Cardiocerebrovascular System

#### 4.1.1. Coronary Heart Disease

The “World Health Statistics 2021 Report” issued by the WHO indicates that cardiovascular diseases remain the top cause of death globally, accounting for 32% of all deaths worldwide [10]. In China, cardiovascular and cerebrovascular diseases (CVDs) are also ranked top as the number one cause of death among the population. The incidence and prevalence of CVDs have continued to rise with the aging of the population and the accelerating pace of urbanization [68]. Among these, coronary heart disease (CHD) is one of the most common CVDs. CHD, also known as coronary atherosclerotic heart disease, is a heart disease caused by coronary artery sclerosis, leading to impaired circulation of the blood supplying the heart, causing myocardial ischemia and hypoxia, and often accompanied by angina pectoris, myocardial infarction, cardiac arrhythmia, and heart failure [69]. Its clinical uses and pharmacological studies have shown that SLR has good therapeutic efficacy for CHD. It can dilate coronary artery blood vessels, increase blood flow, and be used for sedation and analgesia. Ferulic acid, wallichilide, and perlolyrine in SLR may serve as the primary constituents for treating CHD. Von Willebrand factor, coagulation factors, superoxide dismutase 1, and nitric oxide synthase 2 are among the 31 key targets for CHD treatment. SLR may primarily address CHD through anticoagulation, the promotion of angiogenesis, vasodilation, and blood pressure regulation [70].

SLR and Red Peony Root (*Chishao*) are commonly used to activate blood circulation and remove blood stasis. In the practice of Chinese medicine, their combination has significant clinical efficacy. The results of pharmacological experiments have indicated that SLR and Red Peony Root can significantly reduce the triglyceride and low-density lipoprotein cholesterol levels in mice, as well as platelet endothelial cell adhesion molecule-1 expression, neovascularization within plaques, plaque size, and the lipid content within plaques. The pair of SLR and Red Peony Root (*Chishao*) was able to differentially regulate coronary vascular neovascularisation, promote ischemic myocardial neovascularisation by regulating the Notch pathway, reduce infarct size, and improve cardiac function [71]. It also inhibits neovascularisation within atherosclerotic plaques [72]. Yin et al. investigated the molecular mechanism of the NO_3_-NO_2_-NO pathway in the treatment of coronary heart disease. It was found that under hypoxic conditions, SLR significantly reduced the cellular levels of NO_3_ and increased the cellular levels of NO_2_ and NO, thereby achieving vasodilatory and therapeutic effects on CHD [73]. Li et al. analyzed the mechanism of the differences between using the cortex and the pith of SLR to treat coronary artery disease by network pharmacology, combined with SPME-GC-MS. They found the bioactive compounds in the SLR cortex for treating CHD might be carotol, epicubenol, fenipentol, and methylisoeugenol acetate, while 3-undecanone, (*E*)-5-decen-1-ol acetate, linalyl acetate, and (*E*)-2-methoxy-4-(prop-1-enyl) phenol were found to play an important role in the medulla [74]. SLR alkaloids can also significantly dilate the coronary arteries, increase coronary blood flow, and lead to an increase in myocardial oxygen supply, thus treating coronary angina [42]. Further studies on the treatment of coronary heart disease with single components from SLR showed that compounds such as ferulic acid, senkyunolide A, and tetramethylpyrazine had good therapeutic efficacy. They were even more effective when used in combination. Shen et al. conducted a systematic evaluation and meta-analysis of sodium ferulate in the treatment of CHD. It was found that using sodium ferulate in combination with conventional medication such as atorvastatin and nitroglycerin was more efficacious than single conventional therapy [75]. Lei et al. also found the combination of tetramethylpyrazine with senkyunolide A exerted a better vasodilatory function than either component alone [76]. Therefore, SLR is primarily utilized for the treatment of CHD according to vasodilation and enhanced blood circulation.

#### 4.1.2. Cerebral Hemorrhage

Cerebral hemorrhage, commonly known as stroke, is the second leading cause of death worldwide. Strokes have a variety of causes, resulting in damage to the cerebral blood vessels, causing focal or overall brain tissue damage. It is divided into two types: ischemic stroke and hemorrhagic stroke [77]. Meanwhile, stroke is characterized by high morbidity, disability, recurrence, and mortality rates. It is the leading cause of death in Chinese people [78]. The clinical manifestations of stroke are an abnormal appearance on one side of the body, speech or comprehension impairment, vision loss, headache, and vertigo [79]. According to Chinese medicine, SLR “goes up to the head and eyes” and is good for treating diseases such as stroke and hemiplegia and is a “*qi* medicine in the blood” and a “holy medicine for treating wind” [80]. Among the 200 effective formulas in Chinese medicine for the treatment of stroke, SLR appears 84 times, making it the most frequently used CMM among those for activating blood circulation and removing blood stasis [81]. Zhang et al. used network pharmacology to predict the bioactive components, targets of action, and mechanisms of action of SLR for treating stroke and established a diagnostic model. The results revealed that oleic acid and caffeic acid had binding sites with matrix metalloproteinase 9 (MMP9), phosphatase and tensin homolog (PTEN), and tissue inhibitor of metalloproteinase 1 (TIMP1). The diagnostic model indicated that the Fos proto-oncogene, the AP-1 transcription factor subunit (FOS), matrix metalloproteinase 9 (MMP9), phosphatase and tensin homolog (PTEN), tissue inhibitor of metalloproteinase 1 (TIMP1), and toll-like receptor 2 (TLR2) could be used as blood biomarkers for stroke [82].

SLR is effective in ischemic stroke (IS). Yang et al., through network and experimental pharmacology, found that SLR was mainly related to inflammation, hypoxia, endoplasmic reticulum stress, oxidative stress, angiogenesis, coagulation and platelet activation, angiogenesis, endothelial damage, the negative regulation of endothelial apoptosis, and the inflammatory response after ischemic stroke. Pharmacological experiments showed that SLR could improve the neurobehavioral scores of and protect the neurons of IS rats and regulate the expression of the endoplasmic reticulum stress-related targets glucose-regulated protein 78 [GRP78 (HSPA5)], phospho-protein kinase R-like endoplasmic reticulum kinase [p-PERK (EIF2AK3)], and DNA damage-inducible transcript 3 [CHOP (DDIT3)] [83]. Zeng et al. also demonstrated the therapeutic efficacy of SLR. They found a total of 18 chemical components corresponding to 85 anti-ischemic stroke targets based on UHPLC-MS/MS. Among them, coniferyl ferulate, neocnidilide, and ferulic acid were the key anti-ischemic stroke components. The two most important synergistic effects of the chemical constituents of SLR for treating ischemic stroke were also found to be the prevention of infection and the regulation of blood pressure [84]. These results indicate that SLR has good therapeutic efficacy for brain injury and can protect damaged neurons. Furthermore, neuronal apoptosis can be induced by inflammation, and SLR is capable of targeting the relevant factors responsible for inflammation. Pang et al. quantitatively compared the anti-ischemic stroke effects of pairing SLR and Danshen Root at different ratios. The results showed a combination was more effective than either herb alone. Further predictions indicated that Z-ligustilide was also one of the bioactive compounds effective in treating ischemic stroke according to network pharmacology [85].

Pure Z-ligustilide was able to significantly treat IS. Chi et al. found that the *Z*-ligustilide-treated group experienced a significantly better therapeutic effect than the other treated groups in a mouse model of thromboembolic stroke [86]. Intranasal administration of *Z*-ligustilide prevents cerebral ischemia via the nuclear factor erythroid 2-related factor 2 (Nrf2) and heat shock protein 70 (HSP70) signaling pathways [87]. Ferulic acid has a protective effect on brain tissue damage after IS episodes and induces angiogenesis in human umbilical vein endothelial cells by promoting the expression of vascular endothelial growth factor and platelet-derived growth factor in these cells [88]. Wang et al. showed that ferulic acid protects against acute ischemic stroke injury in rats by inhibiting ischemia-induced excitotoxicity, an inflammatory response, and apoptosis [89].

Ferulic acid significantly reduced the infarct size, neurological deficit score, apoptotic index, and cleaved calpain I and cytochrome C levels in acute ischemic stroke in rats. Meanwhile, ferulic acid significantly increased the levels of phosphorylated Akt, mitochondrial bcl-xl/bax genes, the phosphorylated astrocyte PEA15, and hippocampal calcium-binding proteins and the mitochondrial Bcl-2/Bax ratio [90]. Therefore, Z-ligustilide and ferulic acid are the key components in SLR for treating IS, which play a role in protecting damaged nerves in multiple ways.

#### 4.1.3. Atherosclerosis

Atherosclerosis is the main pathological basis of cardiovascular diseases and is formed under the combined action of many risk factors. Atherosclerosis is a progressive pathological process characterized by the structural or functional impairment of large and medium-sized arterial endothelial cells, increased permeability of the intima, and the accumulation of cholesterol and cholesterol lipids in or beneath the intima [91]. Atherosclerotic plaques develop in four stages: fatty streaks, atherosclerotic plaques, complex atherosclerotic plaques, and clinicopathological clinical complications [92]. The underlying pathophysiological mechanisms of atherosclerosis include endothelial dysfunction, lipid deposition, oxidative stress injury, an immune–inflammatory response, and platelet migration and aggregation [93,94,95,96].

Pharmacological network predictions showed there were 167 relevant therapeutic anti-atherosclerosis targets in SLR. Further network topology analysis found 46 core targets, including calcium-sensitive receptor and mitogen-activated protein kinase 3. SLR affects the development of atherosclerosis according to many aspects of biological processes, molecular functions, and cellular composition. SLR may exert its anti-atherosclerotic effects by modulating multiple metabolic pathways, such as neuroactive ligand–receptor interactions and calcium signaling pathways [97]. Pharmacological studies have shown that SLR has preventive and therapeutic efficacy for treating atherosclerosis by lowering serum cholesterol and low-density lipoprotein levels, reducing the degree of atherosclerosis, and reducing erythrocyte deformability in experimental atherosclerotic home remedies [98]. SLR extract significantly inhibited the conversion of the vascular smooth muscle cells from G1-stage into S-stage progression, thereby dose- and time-dependently inhibiting the proliferation of the vascular smooth muscle cells, a process that correlates with NO production [99].

Yang et al. investigated the anti-atherosclerotic effect of lactone extracted from SLR and its mechanism in apoE-deficient mice. It was found that the lesion sizes of the thoracic segments of the aorta were significantly reduced in these mice. Meanwhile, SLR lactone treatments led to a decrease in serum triglyceride, total cholesterol, and low-density lipoprotein cholesterol contents in mice, as well as decrease in the expression of platelet endothelial cell adhesion molecule-1, intercellular adhesion molecule-1 (ICAM-1), monocyte chemoattractant protein-1 (MCP-1), and nuclear factor-kappa B (NF-κB) in atherosclerotic plaques. Furthermore, lactone from SLR dose-dependently attenuated the expression of intercellular adhesion molecule-1 and vascular cell adhesion molecule-1 (VCAM-1) in tumor necrosis factor-α (TNF-α)-stimulated human umbilical vein endothelial cells [100]. Lei et al. found that senkyunolide A and Z-ligustilide ameliorated atherosclerosis and modulated immune autoimmunity by inhibiting the expression of activating protein-1 (AP-1) and NF-κB [101]. Other studies have demonstrated that ferulic acid significantly alleviates atherosclerosis and regulates lipid levels in mice. Its mechanism may be the regulation of the intestinal flora and lipid metabolism through the adenosine 5‘-monophosphate-activated protein kinase α/sterol regulatory element-binding protein 1/acetyl coenzyme A carboxylase 1 pathway [102]. Further studies have shown that ferulic acid also plays a role in the treatment of atherosclerosis by promoting the production of NO in mouse aortic smooth muscle cells (MOVAS) through the endothelial nitric oxide synthase pathway, inhibiting the platelet-derived growth factor (PDGF)-induced migration and proliferation of vascular smooth muscle cells (VSMCs), and promoting the expression of cyclin-dependent kinase inhibitor 1 [103]. Therefore, SLR mainly exerts anti-atherosclerosis effects by improving lipid deposition and endothelial cell function and also has the function of regulating the autoimmune response and vasodilation. The key bioactive compounds in SLR responsible for treating atherosclerosis are ferulic acid and phthalides.

#### 4.1.4. Hypertension

Hypertension is a phenomenon in which the blood flows at a pressure consistently higher than normal against the walls of the blood vessels. Typical symptoms of hypertension include headache, fatigue, restlessness, arrhythmia, palpitations, and tinnitus. Hypertension is strongly associated with stroke, heart failure, and other cardiovascular diseases [104]. Hypertension is a leading cause of premature death worldwide. More than one billion people suffer from the disease globally [105]. SLR is also a commonly used type of CMM for treating hypertension. In ancient medical cases, the frequency of treating hypertension using SLR reached 31.02% [106].

Pharmacological network predictions have indicated that SLR contains 23 pharmacodynamic components for treating hypertension, of which ferulic acid, tetramethylpyrazine, and Z-ligustilide are the main pharmacodynamic components. Meanwhile, 14 main targets of action were also predicted for treating hypertension, which may be related to the HIF-1 signaling pathway, the calcium signaling pathway, the vascular endothelial growth factor (VEGF) signaling pathway, the phosphatidylinositol 3-kinase-protein kinase B (Akt) signaling pathway, the cyclic guanosine monophosphate-protein kinase G signaling pathway, and the estrogen pathway, respectively [107]. Experimental pharmacological studies showed that ferulic acid improved the structure and function of the heart, blood vessels, liver, and kidneys in hypertensive rats [108]. Clinical trials have also confirmed that ferulic acid significantly improves lipid metabolism, effectively reduces blood pressure to a certain extent, and alleviates the development of prehypertension. Ferulic acid has a safety profile superior to that of simvastatin [109]. Ligustilide significantly reduced blood pressure and lipid levels in atherosclerotic spontaneously hypertensive rats, as well as suppressing angiotensin II-induced migration of the vascular smooth muscle cells and downregulating the expression of the migration-associated proteins cellular myelocytomatosis viral oncogene, matrix metalloproteinase 2, Rho-Associated Coiled-Coil-Containing Protein Kinase 1, Rho-Associated Coiled-Coil-Containing Protein Kinase 2, phospho c-Jun N-terminal kinase, and c-Jun N-terminal kinase [110]. SLR may alleviate the progression of hypertension into atherosclerosis by regulating lipid levels in the blood and inhibiting the thickening of vascular smooth muscle.

### 4.2. The Nervous System

SLR has therapeutic efficacy in moving *qi* and relieving pain. It is a good type of CMM for treating migraines [111]. Pharmacological studies have found that many small-molecule compounds in SLR, such as ligustilide, senkyunolides I and A, ferulic acid, and other compounds, are able to enter the brain and have good neuroprotective effects [112,113]. Yu et al. found that SLR extract had a significant protective effect on hypoxia in microglia cells [114]. Wei et al. found that SLR extract significantly improved rat models with cervical root pain and relieved hydropic degeneration and demyelination of the nerve roots [115]. Qin et al. investigated the antioxidant, anti-aging, and neuroprotective effects of SLR in vitro and in vivo (Caenorhabditis elegans CL4176 and CL2355). It was found that SLR extract possessed protective effects [116]. Wang et al. found that SLR has the ability to protect the neurons by promoting the endogenous proliferation of neuroblasts and the production of neural differentiation factors in rats after ischemia injury. Meanwhile, SLR can be anti-neuroinflammatory [117]. The single compounds ligustilide, senkyunolide I, and senkyunolide H are known to have neuroprotective effects on the nerve cells [118,119,120]. Findings from various experimental models consistently suggest that SLR exerts a protective effect on the nervous system, which further demonstrates its neuroprotective role in brain nerve injury, particularly in the management of conditions such as cerebral infarction. Phthalide compounds play an important role in protecting the nervous system.

In addition, SLR extract has potential therapeutic efficacy in Parkinson’s disease [121]. Xie et al. found that SLR was able to influence 1-methyl-4-phenylpyridinium-induced alterations in the biological behavior of a human neuroblastoma cell line by modulating the miR-23a-3p/SNCA axis [122]. SLR also aids in the treatment of depression. Wu et al. found the volatile oil of SLR could improve the open-field test scores and sugar water preference percentages in chronic unpredictable mild stress (CUMS) depression model rats, reduce the static immobility time and increase the body weight of the rats, and significantly increase the dopamine content, prefrontal lobes, and content of NE in the striatum and hippocampus of the CUMS depression model rats. It was shown that the antidepressant effect of SLR volatile oil might be related to an increase in the NE content in the prefrontal cortex and the striatum and the dopamine content in the hippocampus [123]. Based on pharmacological network predictions and preliminary validation in animal experiments, Li et al. showed the material bases for the antidepressant effect of the drug pair of Cape Jasmine Fruit and SLR were butylphthalide, gardenal-I, and 4-methoxyl-phenethyl-butyl ether [124].

### 4.3. The Respiratory System

SLR can assist in the treatment of lung diseases. It is often used in combination with Danshen Root (*Danshen*) and Chinese Angelica (*Danggui*) in the treatment of asthma, chronic obstructive pulmonary disease, pulmonary fibrosis, and lung cancer [125,126]. Modern research results prove that reduced blood flow, increased blood coagulation, and microcirculation dysfunction are the pathological basis of asthma caused by blood stasis. SLR, as a blood-activating and stasis-eliminating agent, is a commonly used clinical adjunct in the treatment of bronchial asthma in the blood-activating and stasis-eliminating category [127]. Wu et al. found that SLR improved idiopathic pulmonary fibrosis through multi-component, multi-target, and multi-pathway co-action according to pharmacological network predictions [128]. Shi et al. found that *Chuanxiong Kangxian* granules improved bleomycin-induced pulmonary fibrosis and attenuated bleomycin-induced oxidative stress and inflammation [129]. Wang et al. demonstrated that SLR had a certain effect on improving the lung function in rats in a pulmonary fibrosis model. Its mechanism might be related to the inhibition of the transforming growth factor-β (TGF-β1)/Smad signaling pathway [130]. Xu et al. found components such as butylphthalide, Z-ligustilide, and senkyunolide E showed strong affinity with seven core targets, including brain-derived neurotrophic factor, the Fos proto-oncogene, the AP-1 transcription factor subunit, prostaglandin-endoperoxide synthase 2, and mitogen-activated protein kinase 14, by network pharmacological prediction and verified this using in vitro experiments in lung cancer cells [131]. Jiang et al. also demonstrated experimentally that ligustilide regulated the proliferation, apoptosis, and aerobic glycolysis of non-small cell lung cancer (NSCLC) cells through the phosphatase and tensin homolog/protein kinase B signaling pathway [132]. Further experiments showed that Z-ligustilide combined with cisplatin reduced cell viability, induced cell cycle arrest, and promoted the apoptosis of cisplatin-resistant lung cancer cells by reducing the levels of phosphatidylinositol 3,4,5-trisphosphate (PIP3) and inhibiting the protein kinase B’s activation [133].

### 4.4. The Digestive System

SLR can assist in the treatment of intestinal disorders. Huang et al. found that SLR and its formulae had anti-ulcer and anti-gastric mucosal damage effects. Their mechanism may be related to affecting the endogenous prostaglandin E2 (PGE2) content in the astric tissue [134]. Liu et al. found that Orange Fruit (*Zhiqiao*)–SLR reversed acute stress in the form of depression-like behavior induced by 15 min of forced swimming and impaired gastrointestinal activity by modulating the glutamatergic system, the AMPAR/BDNF/mTOR/synapsin I pathway, ghrelin signaling, and gastrointestinal nitric oxide synthase in the gastrointestinal tract [135]. The combination of SLR and Kudzuvine Root (*Gengen*) may play a role in the treatment of ischemic stroke by remodeling the intestinal flora in order to regulate short-chain fatty acids and repair the gut–brain barrier [136]. SLR polysaccharides significantly promoted the expression of antioxidant enzymes and their master regulator peroxisome proliferator-activated receptor gamma coactivator-1 alpha (PGC-1α), resulting in a higher antioxidant capacity in the jejunum and cecum of aged mice [60]. Wu et al. found that SLR decoctions had a protective effect on gastric mucosal injury induced by electrolytic damage to the posterior hypothalamus in rats and improved the microcirculation of the gastric mucosa [137]. The protective effect of SLR on the gastrointestinal tract is mainly seen when it is used in combination therapy. SLR polysaccharides may be the main bioactive components for use in adjuvant therapy of gastrointestinal diseases.

### 4.5. The Urinary System

With continuous research progress, pharmacological experts have recognized that blood stasis is both a pathological product of diabetes mellitus and an important factor leading to the further development and deterioration of diabetes mellitus and accompanies the course of the disease over time. So, the method of activating blood circulation and removing blood stasis is one therapeutic method for diabetic nephropathy [138]. SLR is a commonly used type of CMM for activating blood circulation and removing blood stasis. It is often used clinically in combination with other CMM for the prevention and treatment of diabetes mellitus and its complications [139]. Ethanolic extract of SLR attenuated structural and functional renal damage in an in vivo model of streptozotocin-induced diabetes mellitus. Its mechanism of action may be related to the function of inhibiting oxidative stress and inflammation [140]. Qi et al. found that the phthalides in SLR could improve hyperglycemia-induced diabetic renal dysfunction by enhancing nuclear factor erythroid 2-related factor 2 activation and reducing collagen deposition [141]. These results indicate that SLR could inhibit oxidative stress and reduce the inflammatory response by activating nuclear factor erythroid 2-related factor 2 to treat diabetes. Phthalides are the main bioactive components in SLR for treating diabetes. Another study showed that the early administration of SLR attenuated bleomycin-induced renal fibrosis in rats by stimulating the nuclear factor erythroid 2-related factor 2/HO-1 pathway [142].

Further studies have shown that senkyunolide A and senkyunolide I have a good protective effect on the kidneys. Zheng et al. found that senkyunolide A could inhibit extracellular matrix deposition in renal tissues and improve renal function through down-regulation of the Wnt4/β-catenin signaling pathway. It ultimately improved the pathological process of renal interstitial fibrosis in a unilateral ureteral obstruction rat model [143]. Zhu et al. found that senkyunolide I had anti-inflammatory, anti-endoplasmic reticulum stress, antioxidant, and anti-apoptotic effects. It could alleviate ischemia–reperfusion-induced renal injury [144]. Clinical trials also showed that ferulic acid was effective in the treatment of renal vein thrombosis [145].

In addition, SLR has been shown to have significant therapeutic effects on acute renal failure. Ma et al. investigated the effects of SLR on a glycerol-induced acute renal failure rabbit model and found that SLR increased renal blood flow, had prostaglandin-like effects on renal medulla vasodilation, and thus prevented acute renal failure [146]. Hu et al. showed that the preventive effect of SLR on acute renal failure in rabbits might act partly by inhibiting platelet activation and correcting the dysregulation of the prostaglandin-I-2/thromboxane A2 balance in renal medulla tissues [147]. Li et al. found the combination of SLR and rhubarb might inhibit renal tubular epithelial cell apoptosis and ameliorate acute kidney injury and renal fibrosis by inhibiting p38 mitogen-activated protein kinase (MAPK)/p53 signaling [148]. The anti-inflammatory effect of SLR was also reflected in the inhibition of the renal inflammatory response. Cao et al. found that SLR extract inhibited an angiotensin II-induced renal inflammatory response in rats by downregulating the expression of miR-103a-3p [149]. Ji et al. found that SLR improved renal function and suppressed the inflammatory response in Escherichia coli-infected rats with acute pyelonephritis by inhibiting the interleukin-6/signal transducer and activator of transcription 3 axis [150]. SLR demonstrates significant therapeutic efficacy in renal diseases such as renal failure and nephritis. However, further research is needed to elucidate its mechanism of action and medicinal constituents.

### 4.6. Other Pharmacological Activities

In addition to the pharmacological activities mentioned above, SLR has antioxidant, cartilage protective, and whitening properties. Yan et al. found an aqueous extract of SLR exhibited an excellent free radical scavenging ability in vitro. This extract retarded the senescence of Saccharomyces cerevisiae, an important food microorganism that is sensitive to reactive oxygen species stress, and significantly increased the activities of antioxidant enzymes in Saccharomyces cerevisiae, including superoxide dismutase, catalase, and glutathione reductase, as well as their gene expression [151]. Su et al. found that the external application of SLR powder for the treatment of costochondritis had a good effect [152]. Wang et al. found that 50% ethanol aqueous extract of SLR had strong antioxidant effects and inhibitory effects on mushroom tyrosinase in terms of melanin content and intracellular tyrosinase activity under the action of three stimulants (alpha-melanocyte-stimulating hormone, Forskolin, and 3-isobutyl-1-methylxanthine) and dose-dependently reduced the content of reactive oxygen species in response to ultraviolet stimulation [153].

**Table 1:** Mechanism of pharmaceutical action of chemical components or extracts from Szechwan Lovage Rhizome.

Region of Action	Components/Extracts of SLR	Model Types	Targets/Signal Pathways/Genes	Function	References
Coronary heart disease	SLR and Chishao	Myocardial infarction model in mice	Notch pathway	Reduce infarct size and improve cardiac function	[70,72,73]
		Atherosclerosis model in rabbits	TC, FC	Protect the anti-inflammatory function of high-density lipoprotein, maintaining normal lipid transport function	
	SLR extract	Cardiovascular disease model in mice	NO3-NO2-NO	Relaxes the blood vessels and treats coronary heart disease	
Cerebral hemorrhage	Z-ligustilide	Thromboembolic stroke model in mice	Nurr1, BDNF, CXCR4, SDF1αβ	Short-term incubation with low ligustilide concentrations is neuroprotective and can promote neurogenesis	[86,87]
		MCAO model in rats	Nrf 2, HSP 70	Prevents cerebral ischemia	
Atherosclerosis	SLR extract	Atherosclerosis model in rabbits	TG, HDL-C, TC	Reduces serum cholesterol and LDL levels and improves erythrocyte deformability	[98,99,100,101,102,103]
	SLR extract	Rat vascular smooth muscle cell proliferation model	NO	Inhibits the proliferation of the vascular smooth muscle cells	
	SLR lactone	Mouse atherosclerosis model	NF-κB	Reduces serum triglycerides, total cholesterol, and LDL cholesterol	
	Senkyunolide A Z-ligustilide		AP-1, NF-κB	Ameliorates atherosclerosis and modulates autoimmunity	
	Ferulic acid		AMPK α, SREBP1, ACC1	Alleviates atherosclerosis and regulates lipid levels in mice	
	Ferulic acid	Vascular smooth muscle cell proliferation model	NO, p21	Increases the level of NO and induces the migration and proliferation of vascular smooth muscle cells (VSMCs)	
Hypertensive	Ferulic acid	Atherosclerotic and spontaneously hypertensive model in rats	ROS, ALT, AST, ALP	Improves the structure and function of the heart, blood vessels, liver, and kidneys	[108,110]
	Ligustilide	Hypertensive model in rats	c-Myc, MMP2, ROCK1	Reduces blood pressure and lipid levels	
Nervous system	SLR	Microsphere-embolized (ME) cerebral ischemia model in rats	DCX, NeuroD1, GAP-43, GFAP, IL-1β, and TNF-α	Protects the neurons, anti-neuroinflammation	[116,118,119,120,121,122,123]
	Ligustilide, senkyunolide I, and senkyunolide H	Glucose deprivation (OGD) model in mice, intracerebral hemorrhage model cells	MAPK pathway, PI3K-AKT-CREB pathway	Protect the nerve cells	
	SLR extract	Parkinson’s syndrome model in rats	DA, miR-23a-3p/SNCA	Potential efficacy in Parkinson’s disease	
	Volatile oil of SLR	Chronic unpredictable mild stress (CUMS) depression model in rats	DA, NE	Antidepressant	
Respiratory system	SLR–Danshen–Danggui	Lipopolysaccharide-induced acute lung injury model in mice	TNF-α, IL-6, IL-1β, iNOS, COX-2, CRP, IL-6, MCP-1	Inhibit the production of inflammatory factors and treat acute lung injury	[125,129,130,131,132,133]
	SLR	Pulmonary fibrosis model in rats	Tumor necrosis factor-α, interleukin (IL)-1β, and IL-6	Improves pulmonary fibrosis and attenuates oxidative stress and inflammation	
			TGF-β1/Smad pathway	Improves lung function and inhibits pulmonary fibrosis	
	Z-ligustilide	Human NSCLC cell lines H1299 and A549	PTEN/AKT pathway	Regulates the proliferation, apoptosis, and aerobic glycolysis of non-small cell lung cancer (NSCLC) cells	
		Cisplatin-resistant lung cancer cells	PLPP1, AKT, PIP3	Reduces cell viability, induces cell cycle arrest, and promotes apoptosis of cisplatin-resistant lung cancer cells	
Digestive system	SLR	Gastric ulcer model and gastric mucosal injury model in rats	Prostaglandin E2 (PGE2)	Anti-ulcer and anti-gastric mucosal damage effects	[134,135,136,137]
	SLR–Orange Fruit (*Zhiqiao*)	CUMS model in rats	AMPAR/BDNF/mTOR/synapsin I pathway	Reduce depression-like behavior and improve gastrointestinal activity	
	SLR–Kudzuvine Root (*Gegen*)	Middle cerebral artery occlusion (MCAO) model in rats	Claudin-5, ZO-1	Regulate the intestinal barrier	
	SLR decoctions	Gastric mucosal injury model rats	Specific viscosity in the blood and plasma cortisol levels	Protect from gastric mucosal injury and protect microcirculation in the gastric mucosa	
Urinary system	Ethanolic extract of SLR	A streptozotocin (STZ)-induced DN C57BL/6 mice model	Nrf2, NF-κB	Attenuates structural and functional renal damage	[140,141,142,143,144,148,149,150]
	Phthalides in SLR			Improve hyperglycemia-induced diabetic renal dysfunction	
	SLR	Renal fibrosis model in rats	Nrf2/HO-1 pathway	Relieves the degree of renal fibrosis	
	Senkyunolide A	Unilateral ureteral obstruction model in rats	Wnt4/β-catenin signaling pathway	Inhibit extracellular matrix deposition in renal tissues and improves renal function	
	Senkyunolide I	Renal ischemia–reperfusion model in mice	TNF-α, IL-6, Nrf2, HO-1, NQO1, GRP78, CHOP	Alleviates ischemia–reperfusion-induced renal injury	
	SLR and rhubarb	Acute kidney injury in rats	p38 mitogen-activated protein kinase (MAPK)/p53 signaling	Inhibit renal tubular epithelial cell apoptosis and ameliorate acute kidney injury and renal fibrosis	
	SLR	Renal inflammatory model in rats	miR-103a-3p	Inhibits the renal inflammatory response	
		Acute pyelonephritis (APN) model in rats	IL-6/STAT3 axis	Improves renal function and suppresses inflammatory response	

## 5. Evaluation of Pharmaceutical Activities Based on SLR’s Action in Activating Blood Circulation and Removing Blood Stasis

SLR is an important type of CMM with a wide range of clinical uses. SLR’s quality affects the efficacy of Chinese medicine and the quality of related products. Numerous studies have been conducted on the quality of SLR. Currently, SLR’s quality is mainly assessed according to its chemical constituents, including the amounts of single or multiple chemical components, or the correlation between the fingerprints of its chemical components and a specific pharmacological effect [154]. Since most of the single compounds in SLR are unstable, or their correlation with SLR’s efficacy is weak, these research achievements have not been formally applied within the quality standards for SLR. Currently, the quality standard for SLR in the *Chinese Pharmacopoeia* still uses its amounts of ethanol-soluble extracts and ferulic acid as markers [1]. Although these markers are not fully specific to and representative of SLR, this method is a compromise.

CMM are characterized by multi-component and multi-target synergistic effects in producing curative and preventive efficacy [155]. Currently, it is difficult to accurately find all bioactive compounds as a marker of quality assessment. CMM only play a pharmaceutical role when they interact with an organism. The intensity of the pharmaceutical activity of CMM directly reflects their therapeutic efficacy. For example, a bioassay method for evaluating the quality of leeches according to their anticoagulant effect and guidelines for this drug bioassay were included in the *Chinese Pharmacopoeia* (2020 edition) [1]. Therefore, it is more direct and effective to assess the quality of SLR by evaluating the intensity of its pharmaceutical activity.

Although SLR has multiple pharmacological effects, we cannot assess SLR’s quality according to all its pharmacological effects. We should focus on the core of SLR’s action and choose highly relevant pharmacological effects for evaluation. SLR’s action is to activate blood circulation and remove blood stasis. It is mainly used in Chinese medicine to cure a variety of blood stasis symptoms. These symptoms correspond to syndromes caused by poor blood flow, or stagnation of the blood flow, or blood overflowing out of the veins and its stagnation in the body. The main symptoms include clinical pain, lumps, bleeding, purple coloration or cyanosis of the face or lips and tongue, and an astringent or choppy pulse. These symptoms are the same as those for thrombosis, embolism, and infarction in modern medicine. They are also key links in the development of coronary heart disease, stroke, tumors, diabetes mellitus, and other major diseases [156]. According to the international guidelines for the diagnosis of blood stasis, when physical and chemical indicators such as blood rheology, hemodynamics, platelet function, coagulation function, fibrinolytic function, and microcirculation are detected as abnormal, it is suggested that the patient may be suffering from blood stasis [157]. Meanwhile, pharmacological studies on SLR have shown that it has a significant effect on improving blood rheology, hemodynamics, and vascular microcirculation, as well as having an anti-platelet aggregation effect [158,159,160,161]. It can be seen that improving hemorheology, hemodynamics, and microcirculation in the blood vessels and anti-platelet aggregation and anticoagulation are the pharmaceutical activities closely related to the blood-activating and stasis-removing action of SLR. By bioassaying the intensities of these pharmaceutical activities and considering the correlation of these pharmaceutical activities with the formation of blood stasis syndrome and their significance, a comprehensive index on its therapeutic efficacy can be calculated and obtained. Then, the comprehensive pharmaceutical activity of SLR can be effectively evaluated to indicate its quality.

### 5.1. Quantification of Improved Blood Rheology

In the early 20th century, the Nordic pathologist Robin Fahraeus discovered the suspension stability and mobility of blood changed during the course of disease and began to explore the flow properties of blood [162]. However, his findings were not widely accepted at that time. It was not until the late 20th century that hemodynamics became a new discipline for the study of the change laws of the macroscopic and microscopic rheological properties of the blood, blood cells, and blood vessels and their application in medicine, with the development of science and technology, an improvement in people’s understanding of the fluidity of blood and its components, and the evolution of the modern concept of fluid dynamics [163,164,165,166,167]. During the development of blood stasis syndrome, the blood shows different states of thickness, viscosity, coagulation, and aggregation, which have a strong correlation with the changing characteristics of blood flow. Clinical trials show that all the rheological indexes of the blood of patients with blood stasis syndrome change [168]. Therefore, hemorheology treats blood as a fluid flowing in vascular tubes. When the blood’s viscosity is abnormally elevated, blood stasis syndrome occurs. This syndrome is mainly manifested as hyperviscosity syndrome in the blood, including changes in plasma viscosity, erythrocyte pressure/volume, erythrocyte aggregation, erythrocyte deformity, platelet aggregation, and other indicators [169].

Zhou et al. investigated the effects of various ratios of SLR to Tall Gastrodia Tuber (*Tianma*) in the formula for *Dachuanxiong* pills on the hemorheology in a rat model of acute blood stasis and on the blood flow velocity in the common arteries in rabbits. They found that both the medium- and high-SLR-dose groups (10 and 20 times the clinical equivalents) had significantly reduced high- and low-cut whole blood viscosity values; a significantly decreased erythrocyte pressure product, plasma viscosity, and fibrinogen volume; and a significantly improved increased peak blood flow velocity and mean increased blood flow velocity [170].

Li et al. investigated the effects of a combination of SLR and Chinese Angelica (*Danggui*) on the hemorheology of rats with acute blood stasis using five times the clinical equivalent. They found that SLR was able to significantly reduce plasma viscosity and the erythrocyte hematocrit. SLR and Chinese Angelica at a suitable ratio were further capable of reducing whole blood viscosity [171,172,173]. Sun et al. further demonstrated a considered combination of SLR and Chinese Angelicae could promote an improvement in blood rheology [174]. The combination of SLR with other CMM which activate blood circulation and remove blood stasis, such as Red Peony Root (*Chishao*), Danshen Root (*Danshen*), and Nutgrass Galingale Rhizome (*Xiangfu*), all enhance the effect of SLR in improving hemorheological indicators [175,176,177].

Du et al. made SLR into a microemulsion dosage form and observed the effects of this microemulsion on the hematological indices in an adrenaline-induced hematochezia rat model. A high dose of the SLR microemulsion (2 mg/kg at a raw dosage) was found to have a significant ameliorative effect on the erythrocyte deformation index and reduce the hematocrit, erythrocyte aggregation, plasma viscosity, and whole blood viscosity [178]. Zheng et al. quantified the blood rheological indexes in a rabbit model of intraosseous hypertension in the upper end of the tibia when using SLR injection. The results indicated that after 3 weeks of treatment with a 20% SLR injection, the blood rheological indexes returned to normal, except for the erythrocytes. There was no significant difference from those of the normal group. It was suggested that the SLR injection was able to improve the abnormal blood rheology under intraosseous hypertension and thus reduce intraosseous hypertension [179]. Further studies have shown that ferulic acid and levistilide A improve hemorheological indices [180,181].

It is concluded that SLR can reduce plasma viscosity, cellular pressure–volume, erythrocyte aggregation, and blood viscosity, as well as improving erythrocyte deformability and other hemorheological indicators. Thus, it has therapeutic efficacy in treating blood stasis syndrome. The mechanism of action of SLR may involve inhibiting the expression of tissue factor (TF) and intercellular adhesion factor-1 (ICAM-1) through the PI3K-Akt signaling pathway, leading to reduced blood and plasma viscosity. SLR also decreases erythrocyte aggregation and improves erythrocyte deformation by inhibiting extracellular Ca2+ influx and intracellular Ca2+ release. The effect on the blood’s properties within SLR’s action of stimulating blood circulation and removing blood stasis can be quantified by bioassays and the changed levels of one or more related indexes: blood viscosity, hematocrit, the erythrocyte sedimentation rate, and erythrocyte deformability. These indexes can reflect the viscosity of the blood at different stages of blood stasis syndrome.

These indicators can be quantified by the following methods: (1) Blood viscosity includes whole blood viscosity, plasma viscosity, serum viscosity, and water viscosity. Whole blood viscosity mainly depends on the number of red blood cells and their properties. Quantifying the level of whole blood specific viscosity, i.e., the ratio of whole blood viscosity to the viscosity of water, can reflect the overall change in blood rheology [182]. (2) The hematocrit is closely related to blood’s viscosity. An increase in the hematocrit improves blood viscosity. So, determination of the hematocrit can be carried out using the Venn diagram method and the capillary method [183]. (3) The rate of erythrocyte sedimentation reflects the degree of aggregation of the erythrocytes. The faster the sedimentation rate, the higher the degree of aggregation of the erythrocytes. It can be measured by the Wintrobe tube method [184]. (4) The deformability of red blood cells is also related to blood viscosity. The greater the deformability of the erythrocytes, the lower the blood viscosity; in the opposite case, the blood viscosity is higher. The deformability of the erythrocytes can be measured using an erythrocyte deformer [185].

### 5.2. Quantification of Improved Hemodynamics

In the late 1950s, the most representative study of blood’s movement was conducted by Womersley and his collaborator McDonald. The simplified model they developed in the course of their study was able to reflect the actual situation of arterial blood flow more realistically [186]. Hemodynamics was created as a new field of study. Hemodynamics takes the flow and deformation of the blood and blood vessels as the object of study and studies the effect of the viscosity of blood and plasma on the body. It mainly studies the blood flow, blood flow resistance, blood pressure, and their interrelationships in order to determine whether the blood circulatory system is normal or not. It is also one of the most important reference indexes for diagnosing cardiovascular diseases [187]. Hemodynamics is closely related to the symptoms of poor blood flow or blood stasis. In clinical manifestations, blood stasis is often accompanied by different degrees of hemodynamic abnormalities, which are mainly manifested as abnormal arterial blood pressure, heart rate, central venous pressure, pulmonary artery occlusion pressure, cardiac output, mixed venous oxygen saturation, and arterial oxygen saturation. Meanwhile, persistent blood flow abnormalities can cause varying degrees of occlusion and stenosis of the blood vessels [157].

SLR is known to improve hemodynamics and is commonly used in clinics to treat ischemic cerebrovascular disease. Wang et al. established a model of experimental atherosclerosis in rabbits using dietary hyperlipidemia and repeated intravenous injection of heterologous serum. They found that SLR significantly reduced the area of atherosclerotic lesions and inhibited platelet aggregation and prevented thrombosis by improving the mean carotid arterial blood flow and mean carotid arterial blood flow velocity [188]. Deng et al. investigated the effects of SLR on the hemodynamics of normal rats. They found that SLR had a significant effect on the maximum rate of increase in left intraventricular pressure and the maximum rate of decrease in left intraventricular pressure. It is therefore suggested that SLR was able to diminish myocardial systolic and diastolic functions. This result has been verified in different animals [189,190,191]. Yan et al. investigated the effects of SLR on left ventricular hypertrophy and myocardial fibrosis in spontaneously hypertensive rats. It was found that SLR blocked the effects of hypertension on plasma angiotensin II levels, NOS levels, the levels of biochemical indices of myocardial fibrosis, and blood pressure and affected NOS by inhibiting angiotensin II production and preventing an increase in blood pressure in spontaneously hypertensive rats [192].

Ferulic acid is one of the bioactive compounds in SLR. Ferulic acid is known to improve hemodynamics. Hu et al. observed the effects of ferulic acid on cardiac hemodynamics. They found that ferulic acid caused a highly significant increase in left ventricular systolic pressure and the maximum rate of change in left ventricular internal pressure ((+dP/dt)max). But it had no significant effect on left ventricular end-diastolic pressure or heart rate. A dose of 0.4 mg/mL ferulic acid had no significant effect on coronary flow, whilst 40 mg/mL of ferulic acid increased coronary flow very significantly [193,194]. Huang et al. observed the hemodynamic indexes of patients with cirrhosis and portal hypertension when treated with ferulic acid through clinical studies. It was found the rate of effectiveness in the ferulic acid group was 92.7%, which was statistically significant compared with the control group. Ferulic acid effectively reduces portal pressure and the endothelin-1 concentration in patients with cirrhotic portal hypertension without affecting portal blood flow. It has superior efficacy in Child Class A and Child Class B patients [195].

The intensity of the effect of SLR on blood flow rate can be reflected by quantifying the magnitude of the changes in the hemodynamic parameters influenced by SLR. One or several indicators can be measured: (1) Quantifying the blood flow of the cardiac coronary arteries, which mainly includes the determination of the coronary blood flow in in vivo animals, the determination of myocardial trophic blood flow, and the determination of coronary blood flow in isolated hearts [196]. (2) Quantifying cerebral arterial blood flow, mainly using electromagnetic flowmeters and Doppler flowmetry [197]. (3) Quantifying renal blood flow, mainly by direct measurement employing electromagnetic or Doppler flowmeters or measuring the renal blood flow using the radionuclide method or renal artery perfusion [198]. (4) Quantifying the flow of the hepatic blood, including using the hepatic portal vein blood volume method with an electromagnetic flowmeter or hepatic clearance measurement [199].

### 5.3. Quantification of Improved Vascular Microcirculation

Microcirculation refers to the blood circulation between the arterioles and venules. It is the site of material exchange between the blood and tissue cells, including arterioles, capillaries, postcapillary microvessels, venules, microveins, and short arteriovenous circuits [200,201,202]. Microcirculation is often impaired in the case of blood stasis syndrome, such as a slow and stagnant microblood flow, intravascular coagulation, microvascular deformation, perivascular blood seepage, microvascular narrowing or occlusion, etc. [203]. SLR has the effect of improving vascular microcirculation. Shi et al. found that SLR improved nailfold microcirculation disorders in ischemic stroke patients by dilating the venules and accelerating blood flow. Meanwhile, SLR extract improved adrenaline-induced mesenteric microcirculation disorders in rats, acute microcirculation disorders in the bulbar conjunctiva in rabbits caused by high-molecular-weight dextran, acute microcirculation disorders in the soft meninges, and adrenaline-induced microcirculation disorders in the auricles of mice [204]. Shi et al. found that ligustilide, the major compound in SLR volatile oil, had an important effect on the microcirculation of the bulbar conjunctiva in rabbits [205].

Quantifying the effect of SLR on improving vascular microcirculation can be carried out using the following indicators: (1) The rate of microvascular flow: This reflects the perfusion status of microcirculation to a certain extent. It can be measured mainly by quantifying the flow rate of red blood cells by velocimetry [206]. (2) Microvascular diameter: The degree of dilation and contraction of the microvessels is determined by changes in the size of their diameter [207]. (3) The number of intersections of the capillary network: This reflects the filling status of the capillaries [208]. Based on these quantitative indicators, the microcirculation in different parts of the body can be determined, such as the auricular microcirculation, buccal sac microcirculation, mesenteric microcirculation, meningeal microcirculation, hepatic microcirculation, tracheal microcirculation, pulmonary microcirculation, renal microcirculation, and cutaneous microcirculation.

### 5.4. Quantification of Anti-Platelet Aggregation

In the process of the formation of blood stasis syndrome, blood stasis forms due to abnormal platelets and excessive aggregation. The main effect of SLR on the platelets is anti-platelet aggregation. Platelet aggregation is one of the main functions of platelets. Thrombin production is initiated by tissue factors in the vessel walls or blood during pathological thrombosis and physiological hemostasis. Platelets aggregate in response to thrombin converting fibrinogen into fibrin, with the concomitant activation of the platelets, or the platelets are activated once exposed to subendothelial collagen after vascular rupture [209,210,211,212]. Pharmacological studies have shown that SLR has significant antiplatelet aggregation effects. Chemical components such as ferulic acid, ligustilide, senkyunolides A, I, and H, and levistilide A effectively inhibit platelet aggregation induced by thrombin, adenosine diphosphate, platelet-activating factor, and reduce the rate of platelet aggregation [41,213,214,215].

Platelet aggregation can be quantified by light transmission aggregometry, resistive antimetry, VerifyNow, Plateletworks, etc. [216,217,218]. Light transmission aggregometry is the most commonly used method in clinical testing. Since platelet-rich plasma has a certain turbidity, when the inducers adenosine diphosphate, collagen, epinephrine, arachidonic acid, thrombin, etc., are added, as a result, platelets aggregate, platelet-rich plasma’s turbidity decreases, and the transmittance increases. The higher the degree of platelet aggregation, the more pronounced the decrease in turbidity. Therefore, the transmittance intensity of platelet-poor plasma is set at 100% aggregation, and that of platelet-rich plasma is set at 0% aggregation, and the change in transmittance is recorded by a platelet aggregometer [156]. So, the rate of platelet aggregation can be calculated.

### 5.5. Quantification of an Anticoagulant Effect

Abnormalities in the coagulation system are the pathological basis for the formation of blood stasis syndrome. When pathological factors activate the coagulation system, the production of thrombin triggers a cascade reaction which catalyzes the formation of fibrin from fibrinogen and the formation of thrombi [209]. SLR has significant anticoagulant effects. Huang et al. found components such as ferulic acid, chlorogenic acid, and isochlorogenic acid A had a strong binding capacity for and inhibitory activity against thrombin using affinity ultrafiltration coupled with HPLC-Q-Orbitrap-MS^n^ and bioactive tests in vitro [219]. Clinically, thrombin time is often used to monitor the common thrombin pathway; activated partial thromboplastin time is used to monitor the endogenous thrombin pathway; and prothrombin time is used to monitor exogenous thrombin pathways. A short clotting time may be seen with the formation of a thrombus, as well as obstructed blood flow [220].

### 5.6. Calculation of a Comprehensive Index of Pharmaceutical Activities

The activating effect of SLR on the blood and blood vessels is comprehensively indicated in it improving blood rheology, hemodynamics, and vascular microcirculation and its antiplatelet aggregation and anticoagulant effects. Any given indicator of pharmaceutical activity only reflects a specific pharmaceutical effect, and a single pharmaceutical effect cannot fully reflect SLR’s action in activating blood circulation and removing blood stasis. Therefore, it is necessary to combine pharmaceutical indexes together and calculate the comprehensive pharmaceutical index to evaluate SLR’s entire action. The values measured for the above for improving hemorheology, improving hemodynamics, improving vascular microcirculation, and exerting antiplatelet aggregation and anticoagulant effects using SLR are first standardized. Then, an expert scoring method is used to set the weighting coefficients for each indicator by peer experts who judge the importance of each pharmaceutical activity indicator for SLR’s action to activate blood circulation and remove blood stasis based on the theories of Chinese medicine and the mechanisms of its pharmacological effects. Finally, a comprehensive index is obtained by calculating these pharmaceutical activities and their weighting coefficients. All of the pharmaceutical activities related to SLR’s activation of blood circulation and removal of blood stasis and its quality are effectively evaluated.

## 6. Conclusions

Although many research works have been conducted on the quality evaluation methods for SLR, the current legal markers for quality assessment of SLR are weak in terms of specificity to SLR and poorly representative of SLR’s medical action, for reasons such as the volatility of the known bioactive compounds resulting in difficulty in their standardization, their instability resulting in insufficient purity, or their wide distribution resulting in a lack of specificity, as is the case for ferulic acid. Thus, it is hard to effectively evaluate and control the quality of SLR. Therefore, new quality evaluation methods for SLR should be further researched and developed for application in practical detection.

The chemical constituents in SLR are mainly phthalides, phenolic acids and their esters, alkaloids, and polysaccharides. Phthalides are the most abundant and characteristic constituents of SLR. Its phthalide components, which have significant pharmaceutical activities related to SLR’s action, are some of the most efficacious components. Phthalides can be divided into two types, i.e., monomeric phthalides and dimeric phthalides. However, the known monomeric phthalides are unstable and are difficult to standardize. None of them have been used as chemical markers to assess SLR’s quality in pharmacopeia or monography. Meanwhile, dimeric phthalides, i.e., levistolide A, are solid at ambient temperature, making them potential chemical markers for assessing SLR’s quality. Therefore, dimeric phthalides are excellent potential compounds as chemical markers of quality in SLR. And it is a future direction to identify suitable dimeric phthalide components as chemical markers for evaluation of SLR’s quality.

When CMM take effect in the body, their therapeutic efficacy can play out. The intensity of their pharmaceutical activity more directly reflects the quality of the CMM. So, the quality of SLR can be assessed by evaluating its pharmaceutical activities. A bioassay is a good choice for assuring the pharmaceutical activity of CMM and clarifying their pharmaceutical components and thus is an effective method for evaluating SLR’s quality, a typical example of CMM containing an unstable volatile composition. SLR has efficacy for diseases of the cardiovascular and cerebrovascular system, the nervous system, the respiratory system, and the urinary system. As regards SLR’s action in activating blood circulation and removing blood stasis, its overall pharmaceutical activities can be evaluated by bioassaying its intensity in improving hemorheology, hemodynamics, and blood microcirculation and exerting anti-platelet aggregation and anticoagulation effects and then setting the weight coefficient for each pharmaceutical activity based on the contribution of the specific activity to SLR’s entire action and calculating a comprehensive index of SLR.

## Data Availability

Data are contained within the article and Supplementary Materials.

## Supplementary Materials

The following supporting information can be downloaded at https://www.mdpi.com/article/10.3390/ph17091157/s1. Figure S1: Chemical composition of Szechwan lovage rhizome; Table S1: Chemical composition of Szechwan lovage rhizome.

## Abbreviations

AngII	Angiotensin II
AP-1	Activating protein-1
CGMP-PKG	Cyclic guanosine monophosphate-protein kinase G
CHD	Coronary heart disease
CMM	Chinese materia medica
CUMS	Chronic unpredictable mild stress
CVDs	Cardiovascular and cerebrovascular diseases
HUVEC	Human umbilical vein endothelial cell vessels
ICAM-1	Intercellular adhesion molecule-1
NF-κB	Nuclear factor-kappa B
LDL	Low-density lipoprotein
MCP-1	Monocyte chemoattractant protein-1
MOVAS	Mouse aortic smooth muscle cells
MAPK	Mitogen-activated protein kinase
NSCLC	Non-small cell lung cancer
PDGF	Platelet-derived growth factor
PGC-1α	Peroxisome proliferator-activated receptor gamma coactivator-1alpha
PGE2	Prostaglandin E2
PI3K	Phosphatidylinositol 3-kinase
PIP3	Phosphatidylinositol 3,4,5-trisphosphate
SLR	Szechwan lovage rhizome
TGF-β1	Transforming growth factor-β
TNF-α	Tumor necrosis factor-α
VCAM-1	Vascular cell adhesion molecule-1
VEGF	Vascular endothelial growth factor
VSMCs	Vascular smooth muscle cells
