# Supplementary material for: Evaluation of the Pharmaceutical Activities of Chuanxiong, a Key Medicinal Material in Traditional Chinese Medicine
Source: Pharmaceuticals (Basel). 2024 Aug 31;17(9):1157. doi: 10.3390/ph17091157 (PMC11434844; doi:10.3390/ph17091157)
Supplement: Supplementary file 1 [file pharmaceuticals-17-01157-s001.zip › pharmaceuticals-3159648-Supplementary materials.pdf]

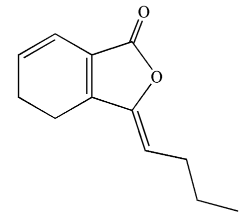

1

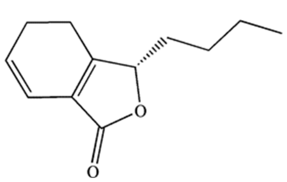

2

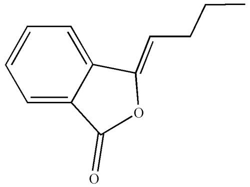

3

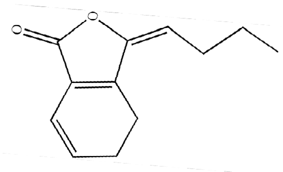

4

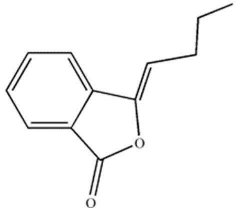

5

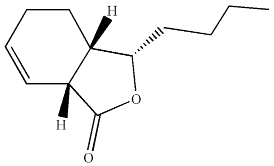

6

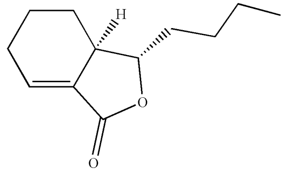

7

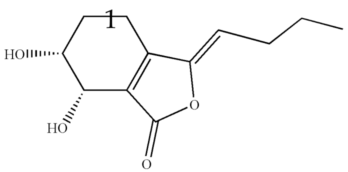

8

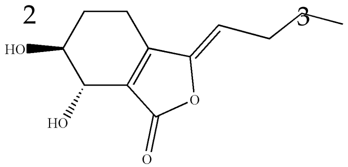

9

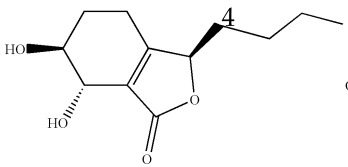

10

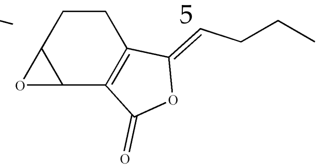

11

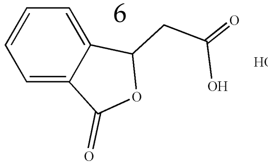

12

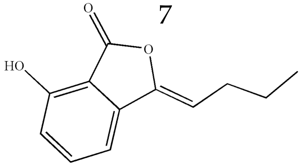

13

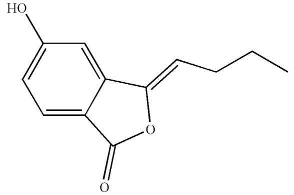

14

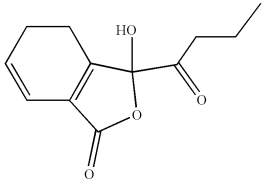

15

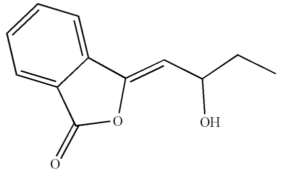

16

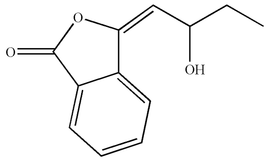

17

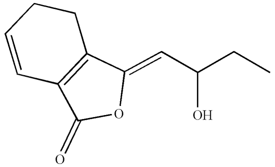

18

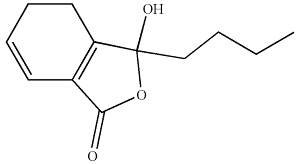

19

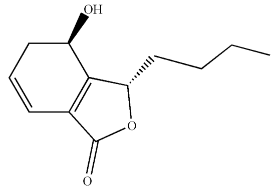

20

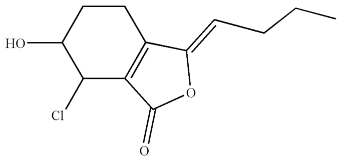

21

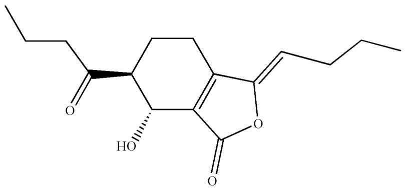

22

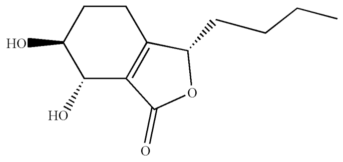

23

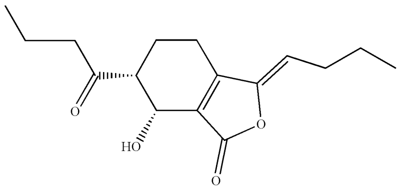

24

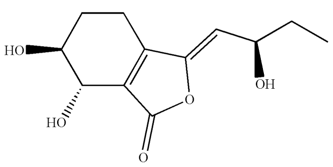

25

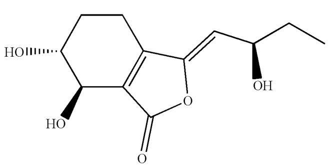

26

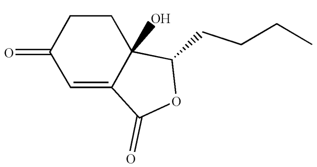

27

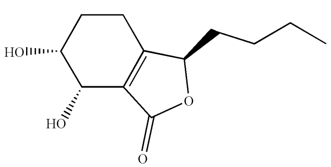

28

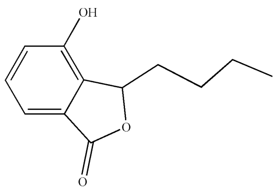

29

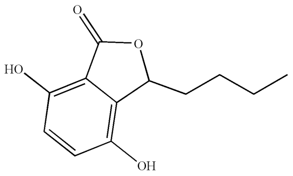

30

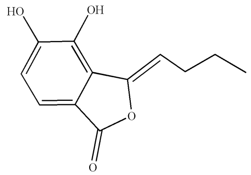

31

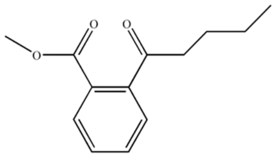

32

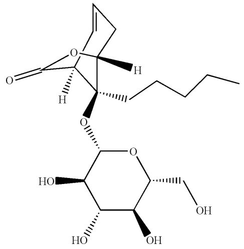

33

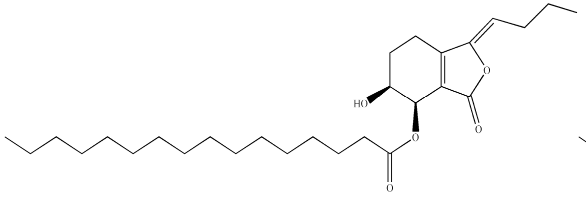

34

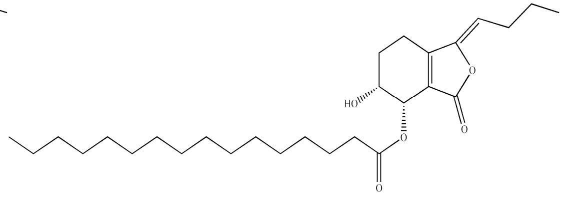

35

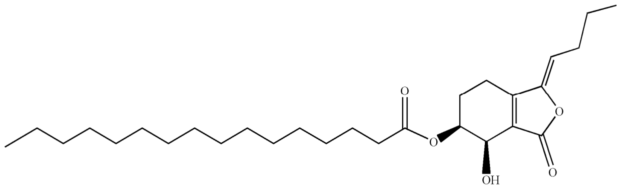

36

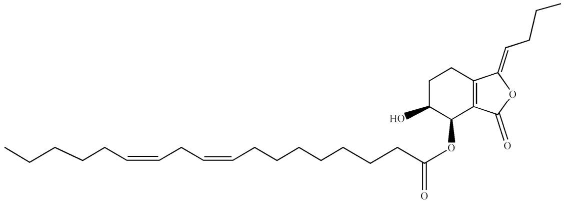

37

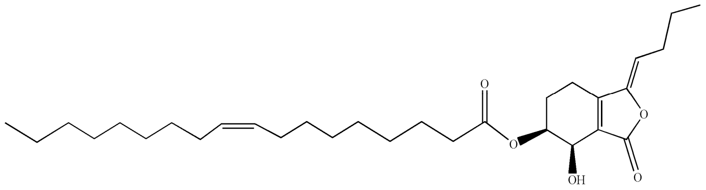

38

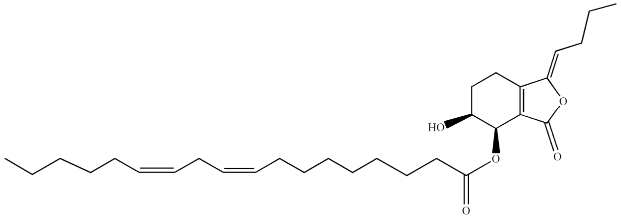

39

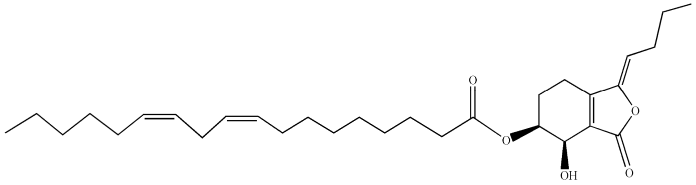

40

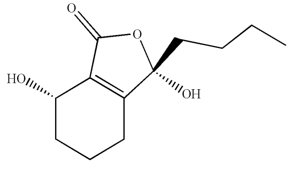

41

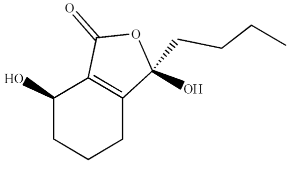

42

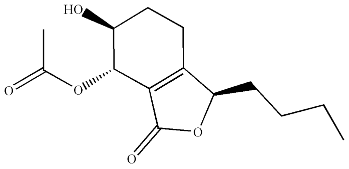
43

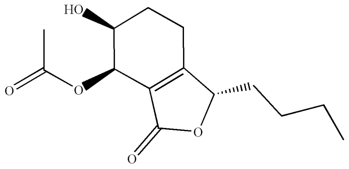
44

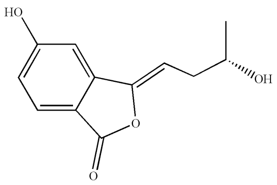
45

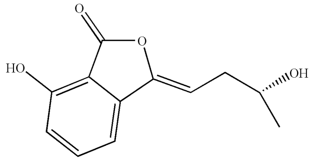
46

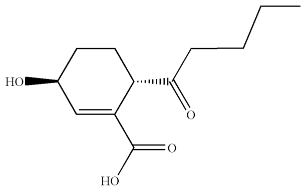
47

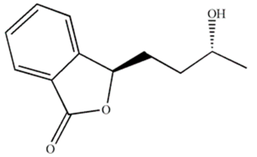
48

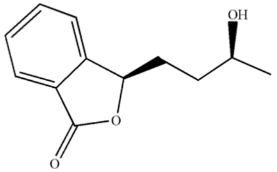
49

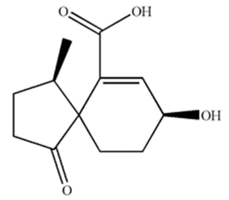
50

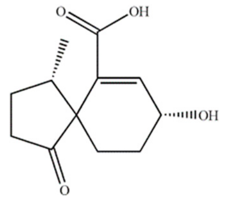
51

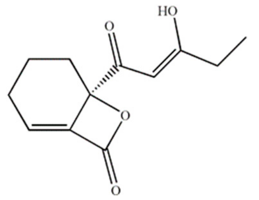
52

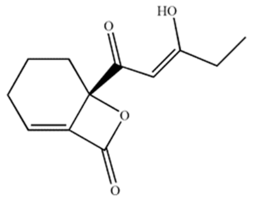
53

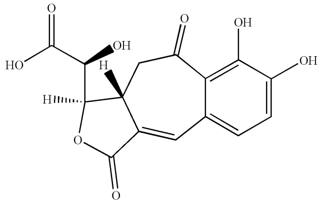
54

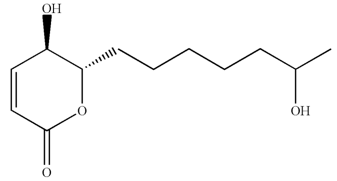
55

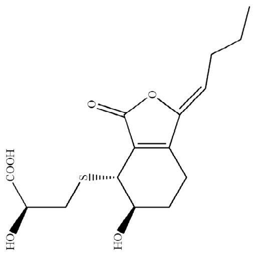
56

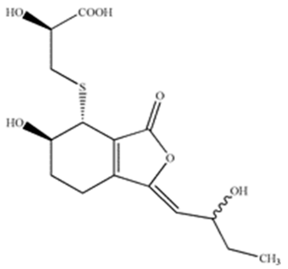
57

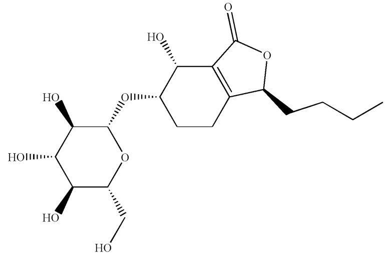
58

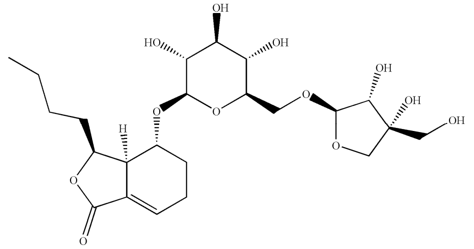
59

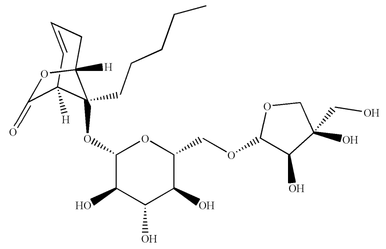
60

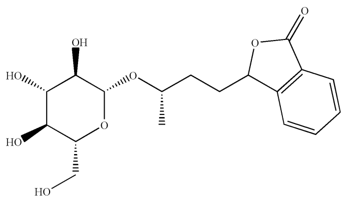
61

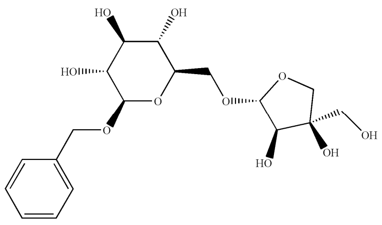
62

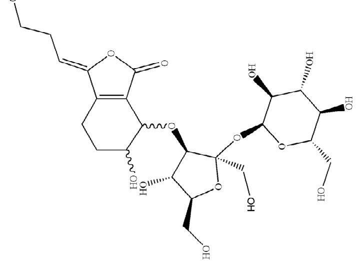
63

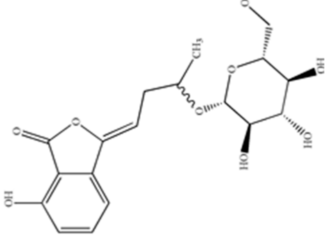
64

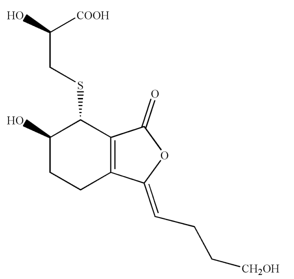

65

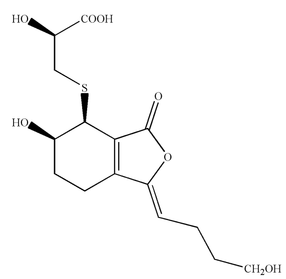

66

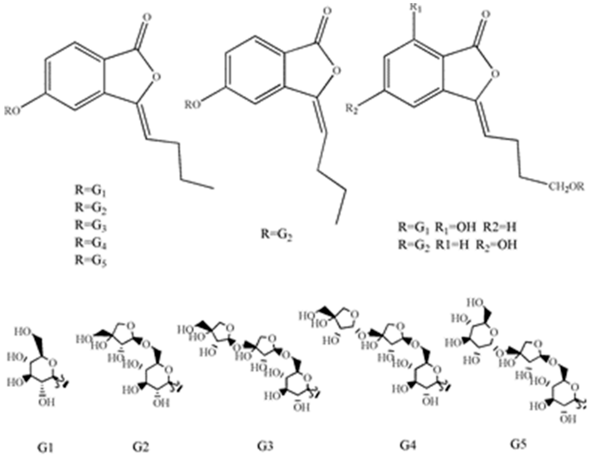

67-

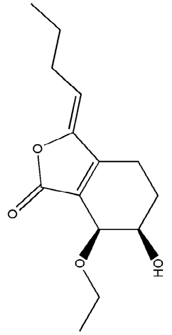

75

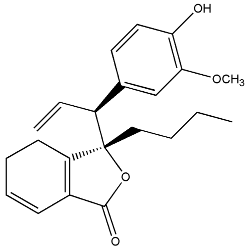

76

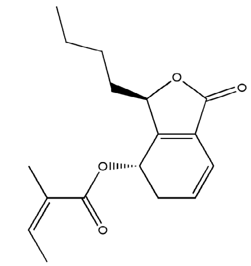

77

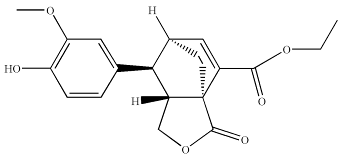

78

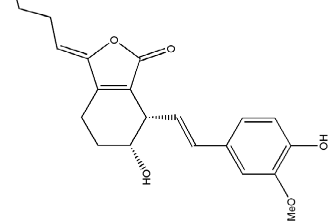

79

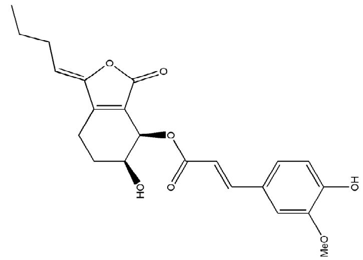

80

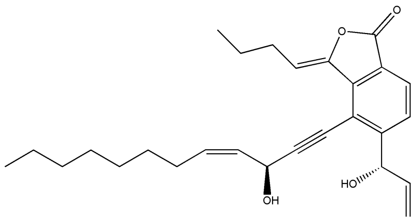

81

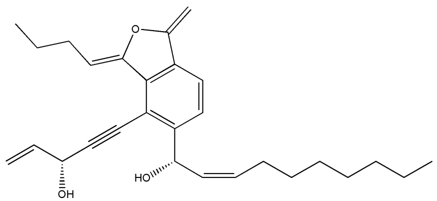

82

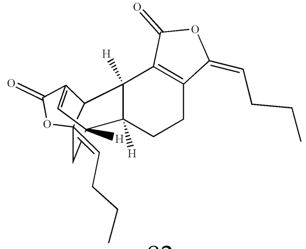

83

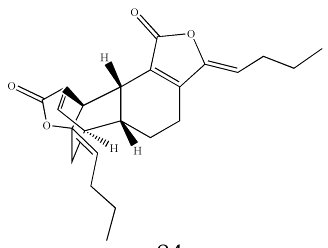

84

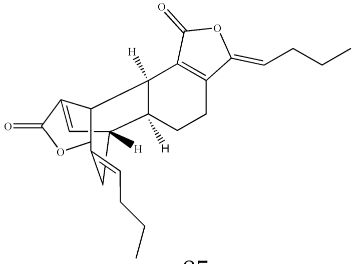

85

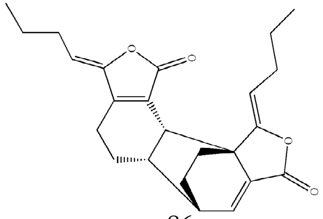

86

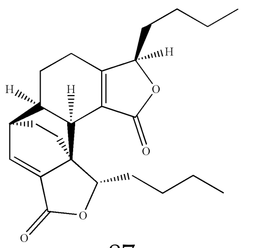

87

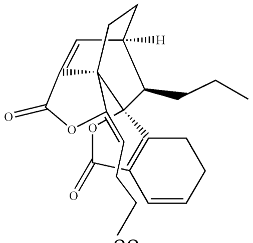

88

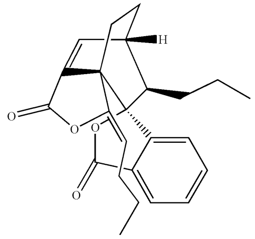

89

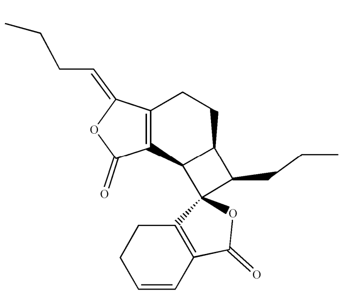

90

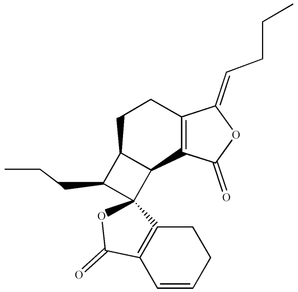

91

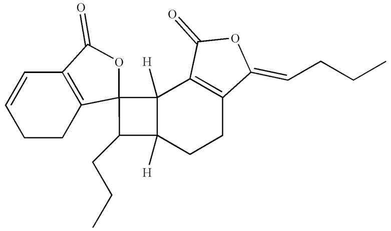

92

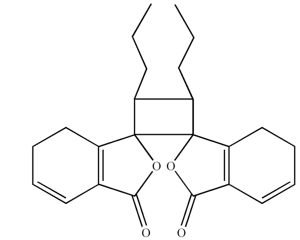

93

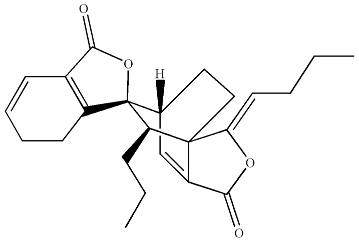

94

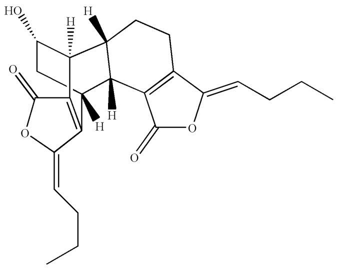

95

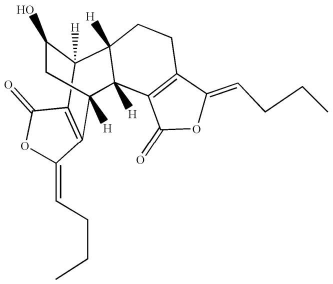

96

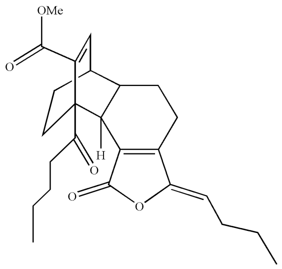

97

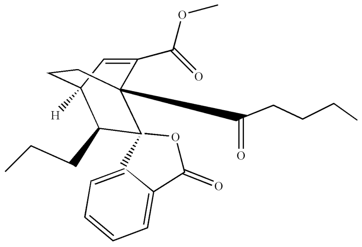

98

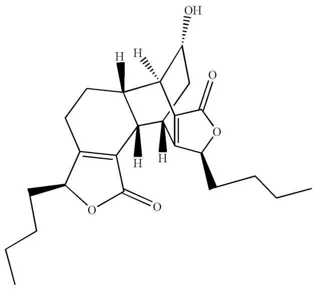

99

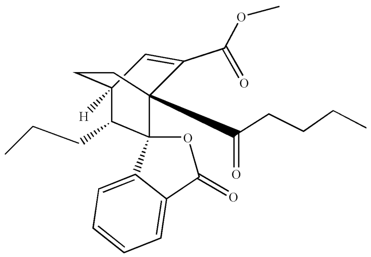

100

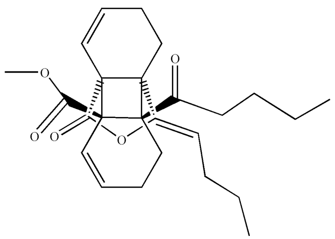

101

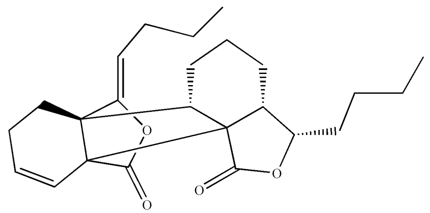

102

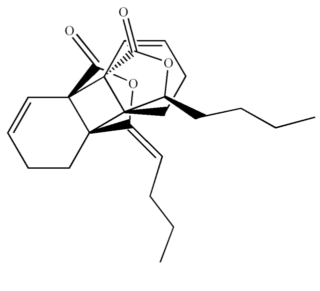

103

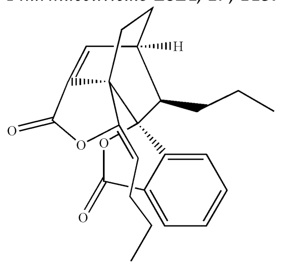

104

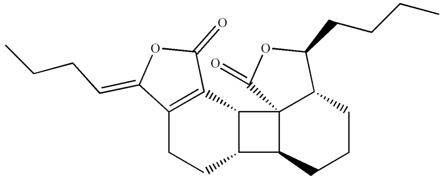

105

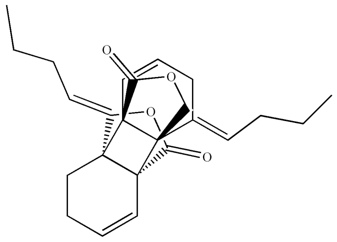

106

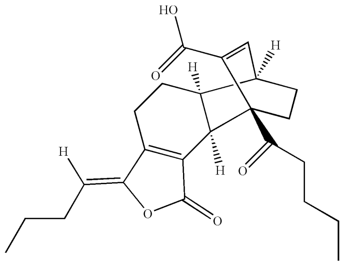

107(+) \ 108(-)

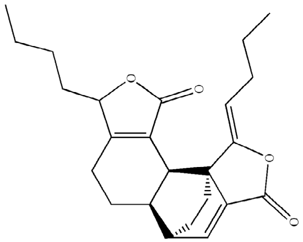

109

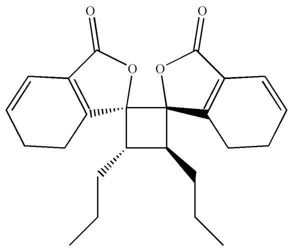

110

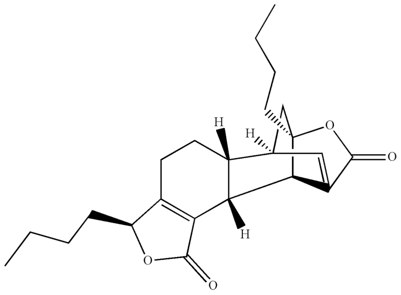

111

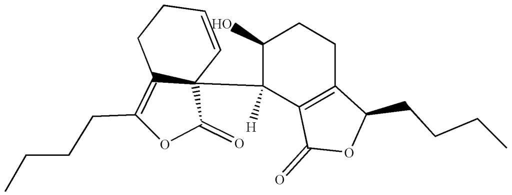

112

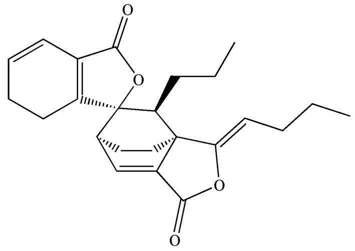

113

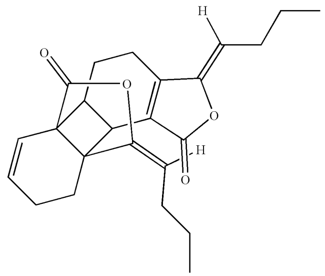

114

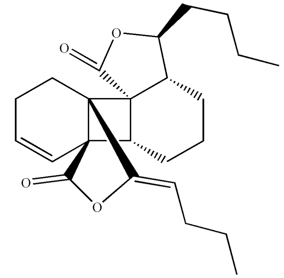

115

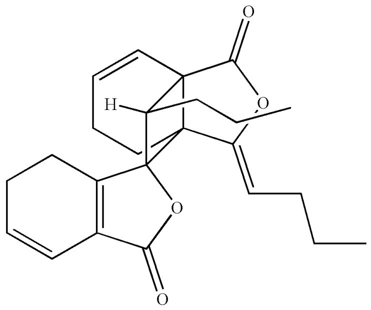

116

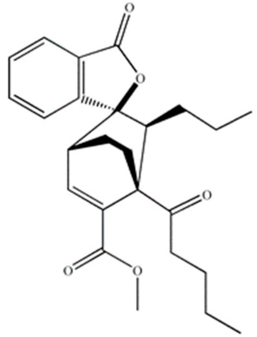

117

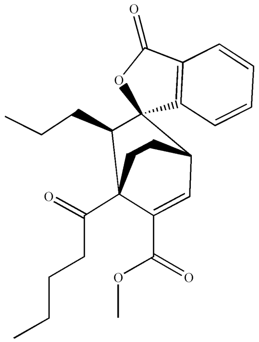

118

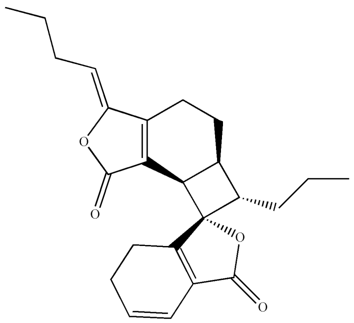

119

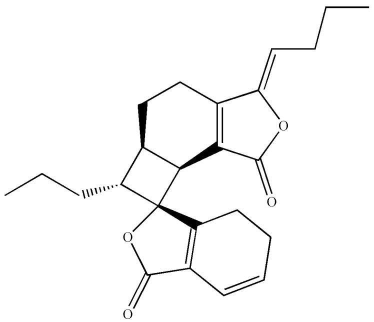

120

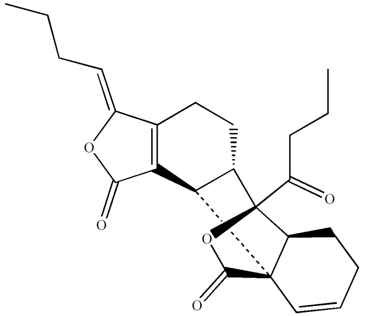

121

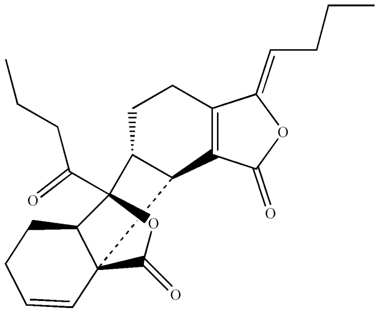

122

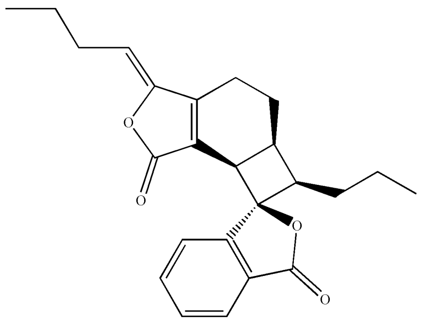

123

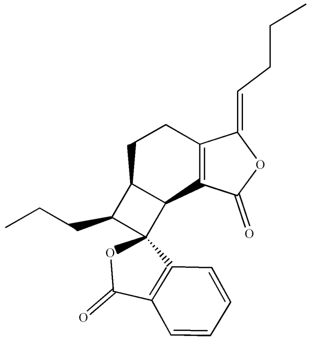

124

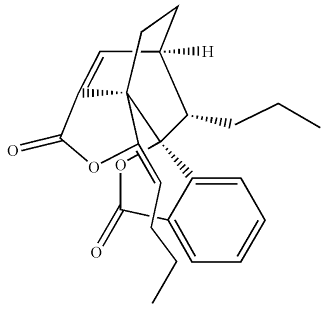

125

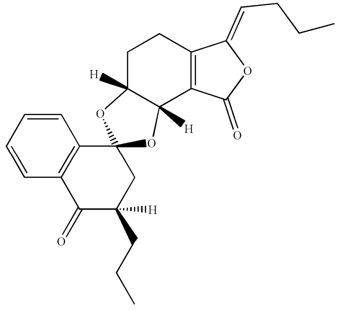

126

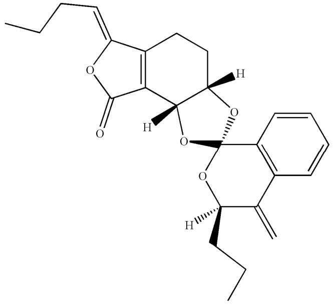

127

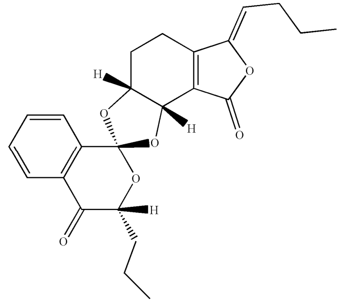

128

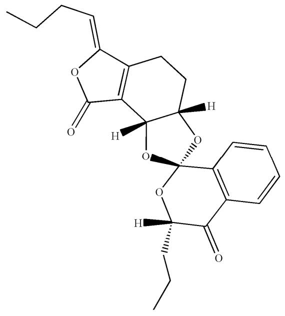

129

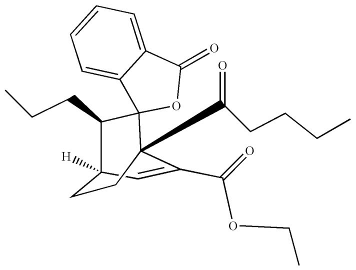

130

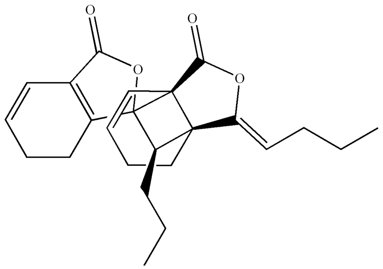

131

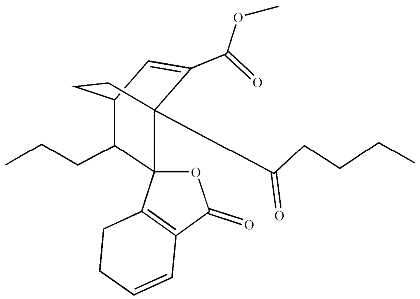

132

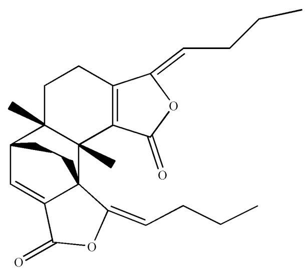

133

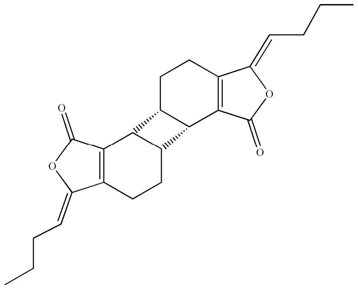

134

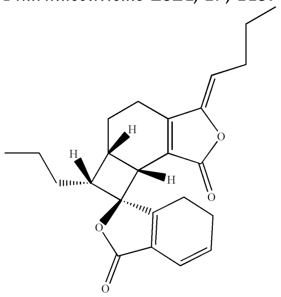

135

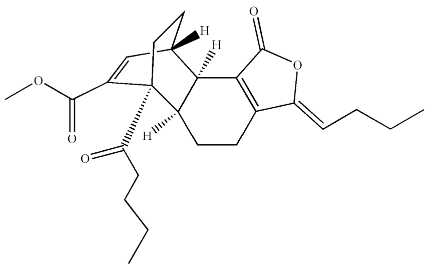

136

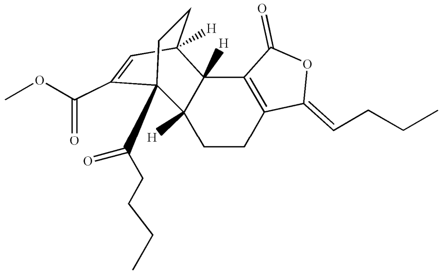

137

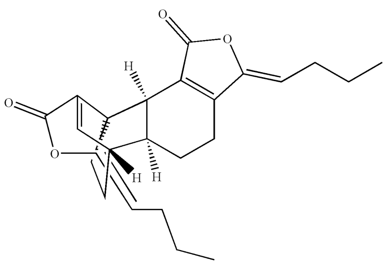

138

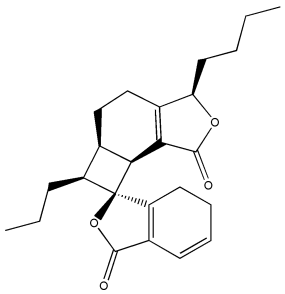

139

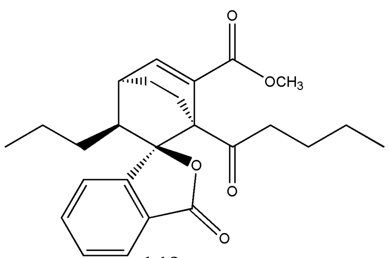

140

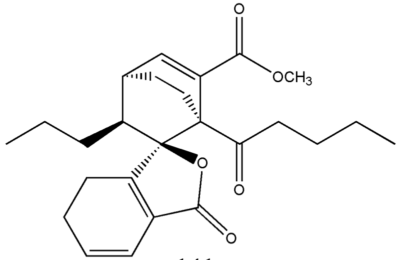

141

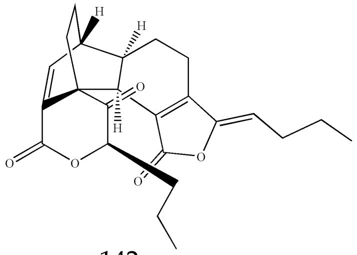

142

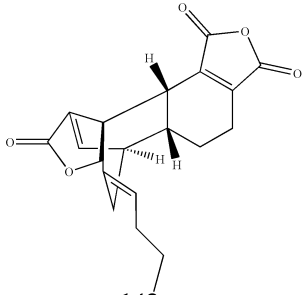

143

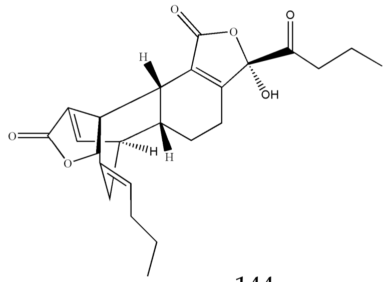

144

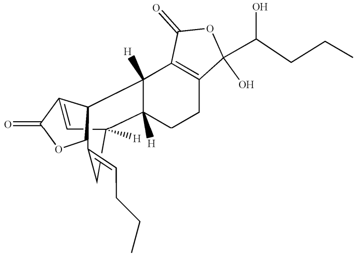

145

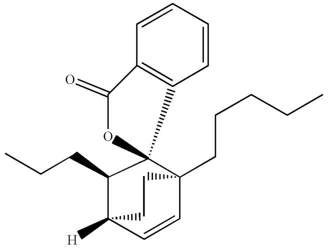

146

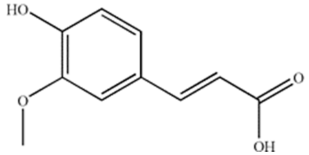

147

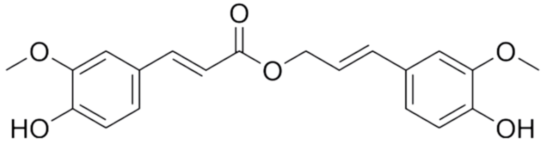

148

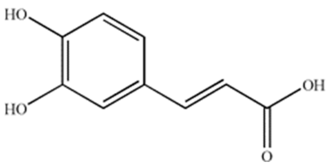

149

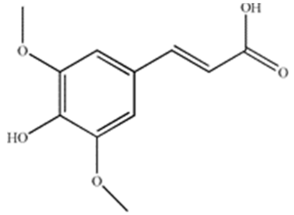

150

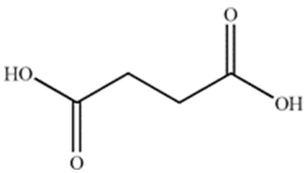

151

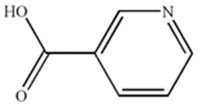

152

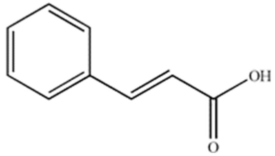

153

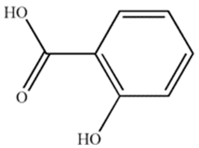

154

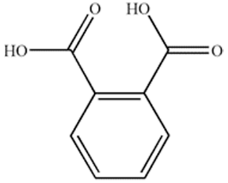

155

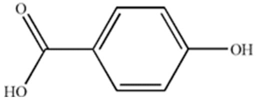

156

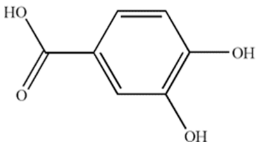

157

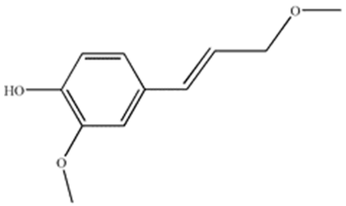

158

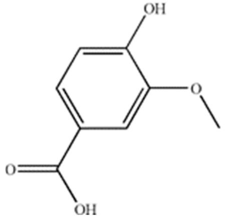

159

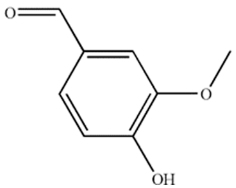

160

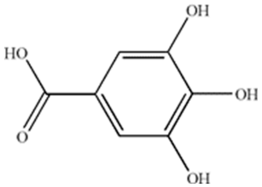

161

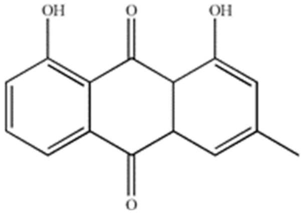

162

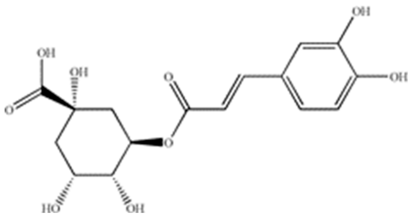

163

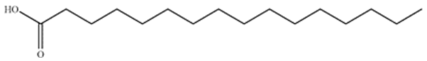

164

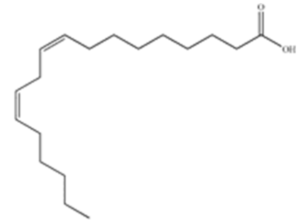

165

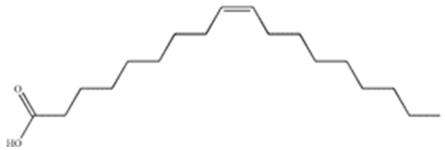

166

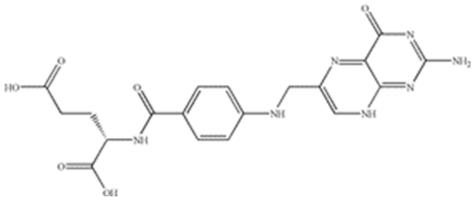

167

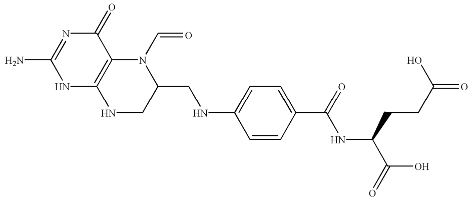

168

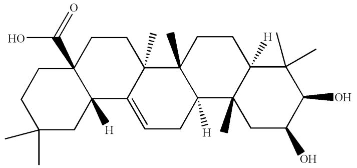

169

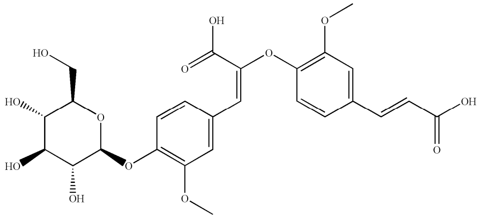

170

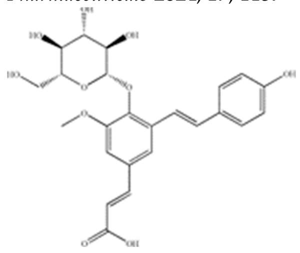
171

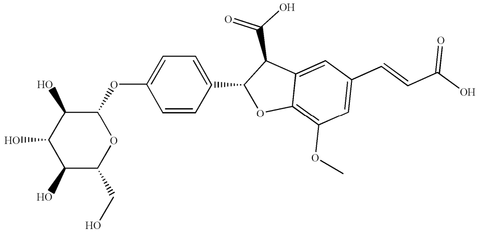
172

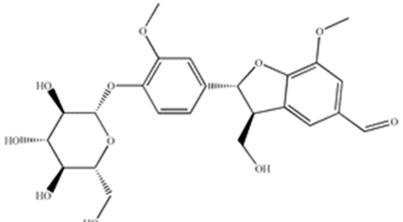
173

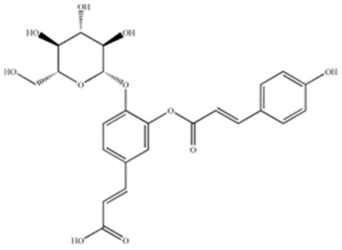
174

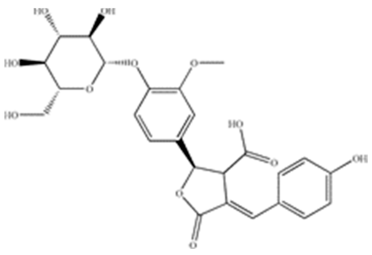
175

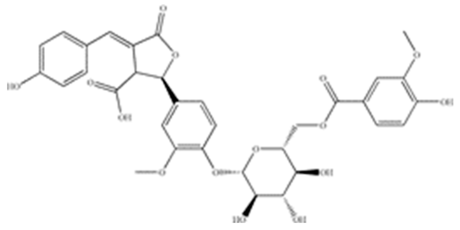
176

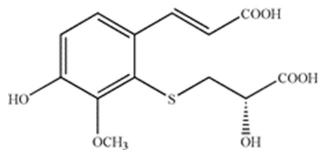
177

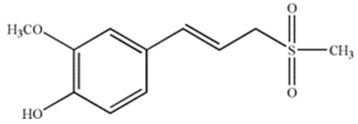
178

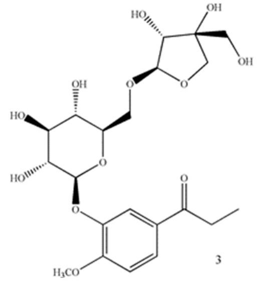
179

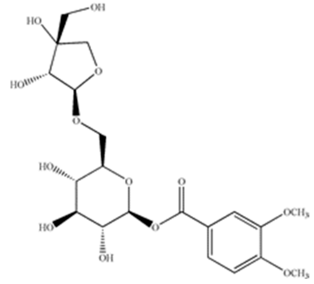
180

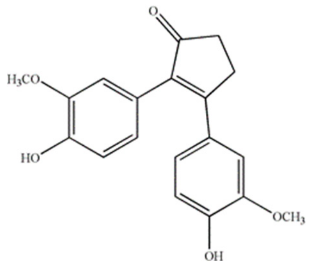
181

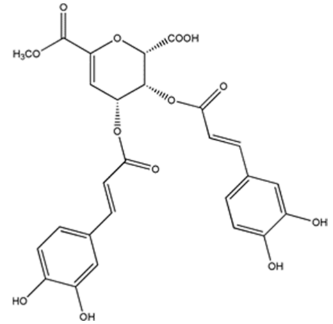
182

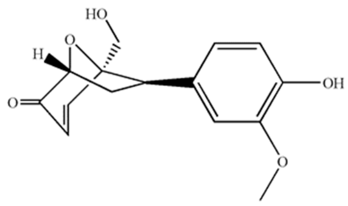
183

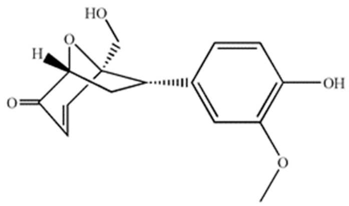
184

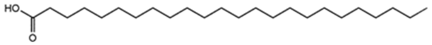
185

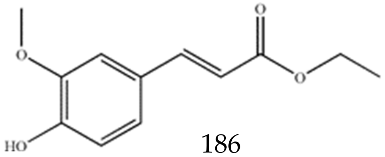
186

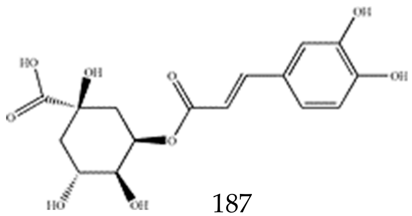
187

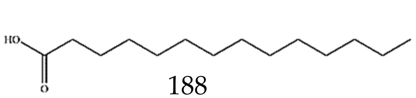
188

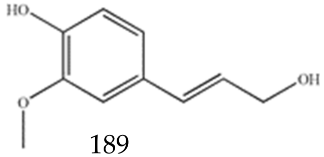
189

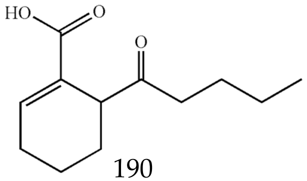
190

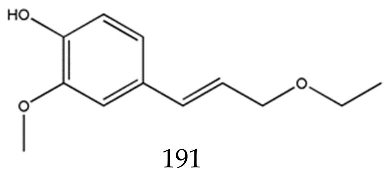
191

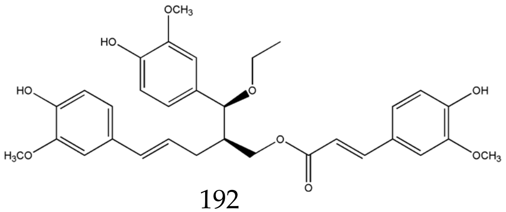
192

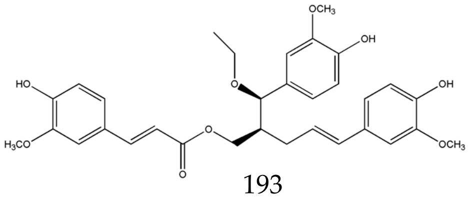
193

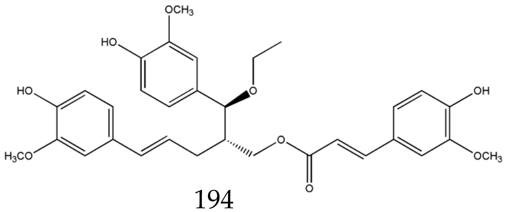
194

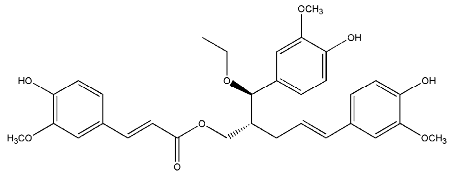
195

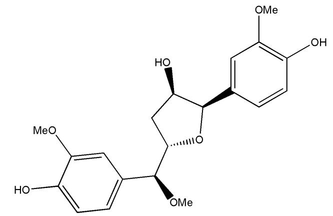
196

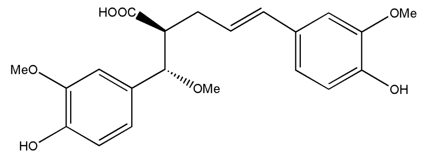
197

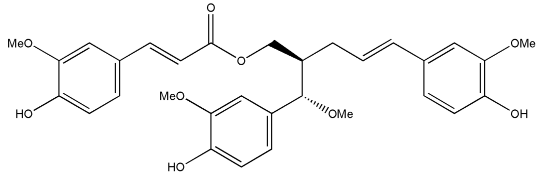
198

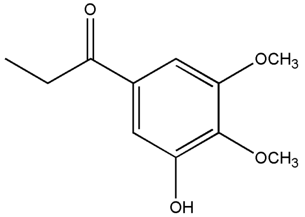
199

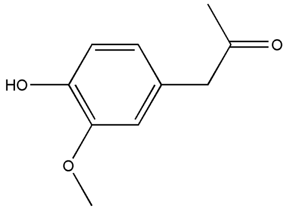
200

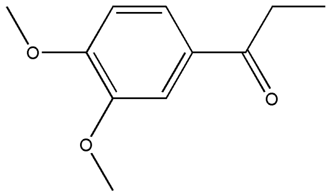
201

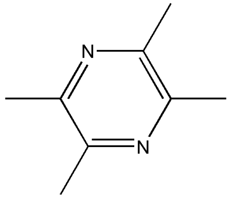
202

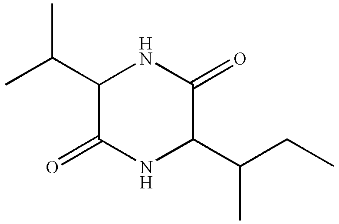
203

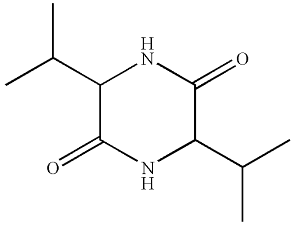
204

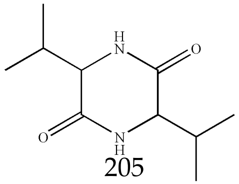
205

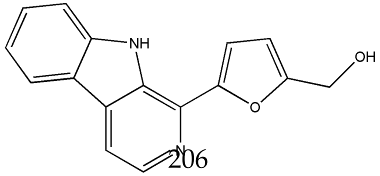
206

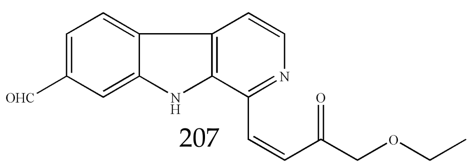
207

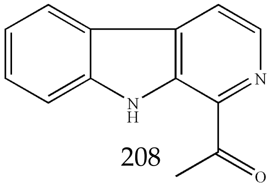
208

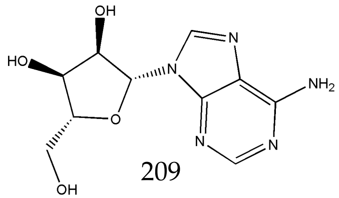
209

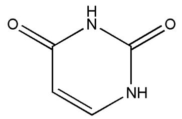

210

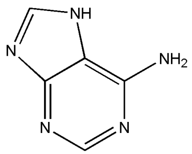

211

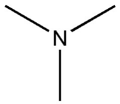

212

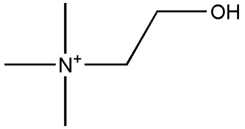

213

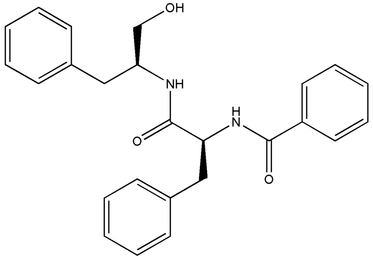

214

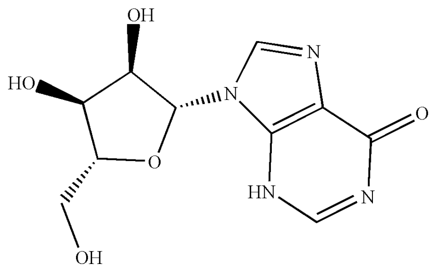

215

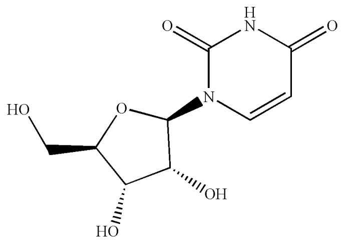

216

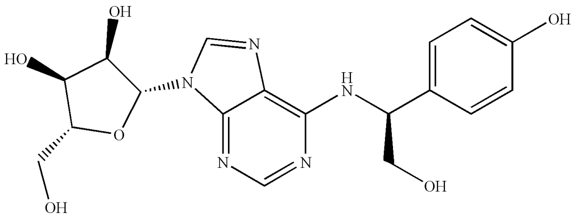

217

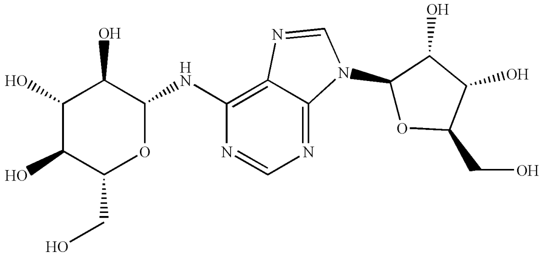

218

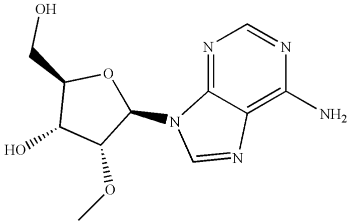

219

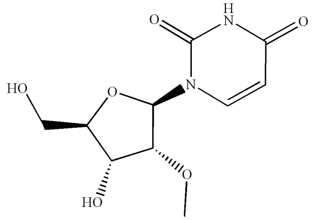

220

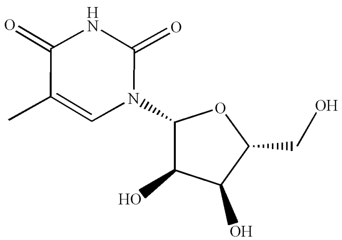

221

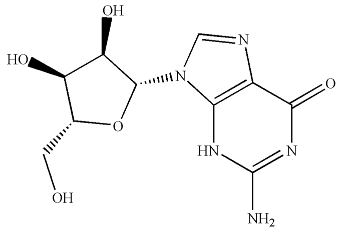

222

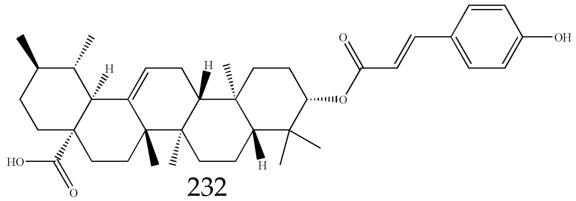

232

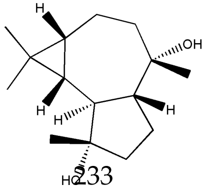

233

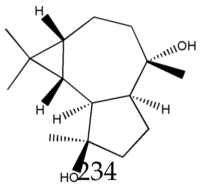

234

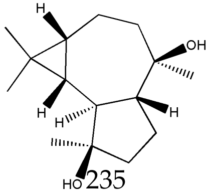

235

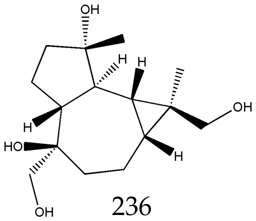

236

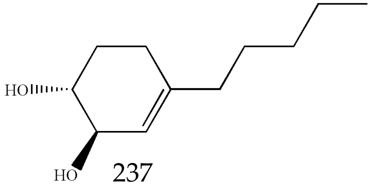

237

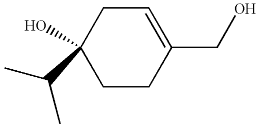

238

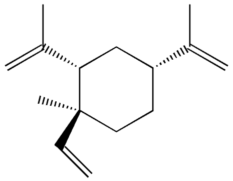

239

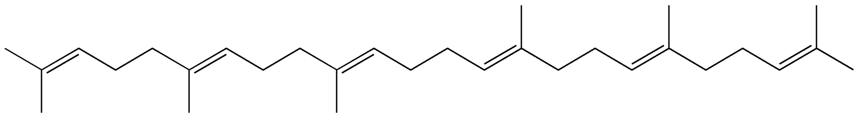

240

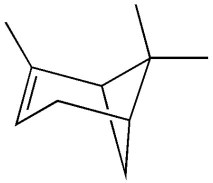

241

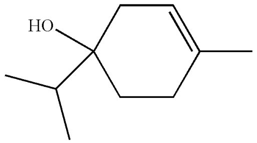

242

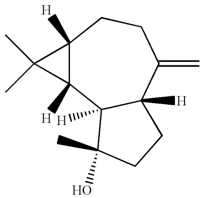

243

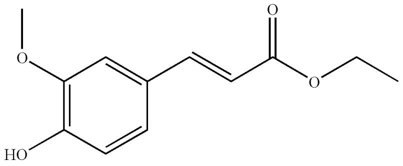

244

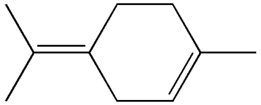

245

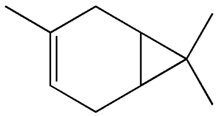

246

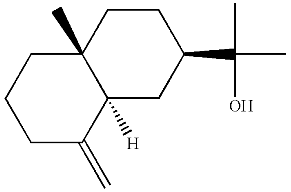

247

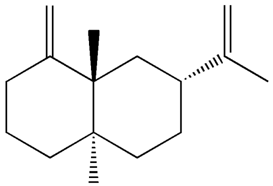

248

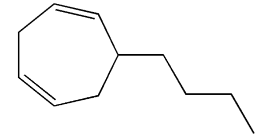

249

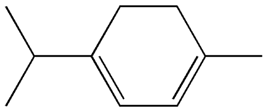

250

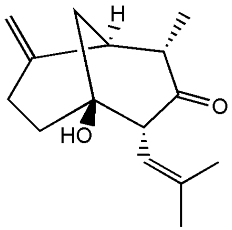

251

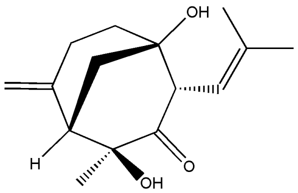

252

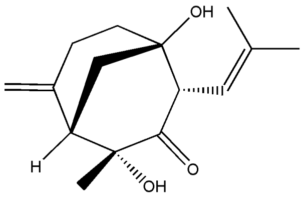

253

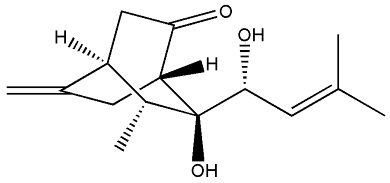

254

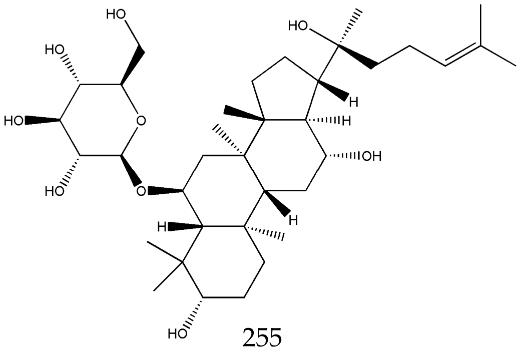

255

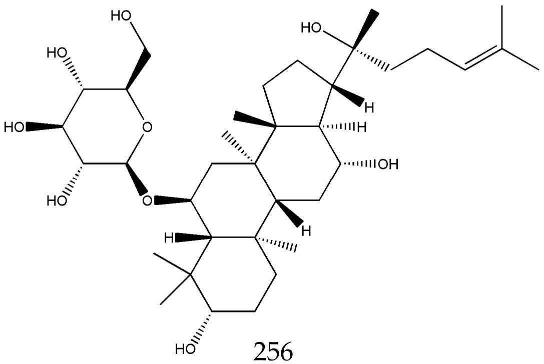

256

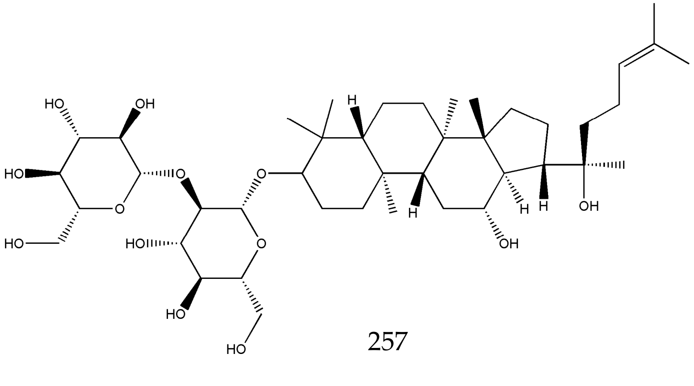

257

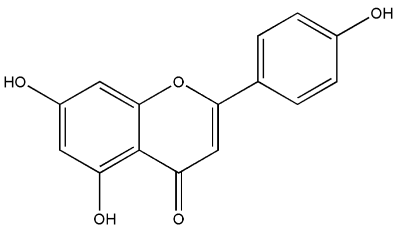

258

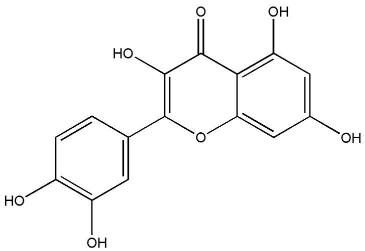

259

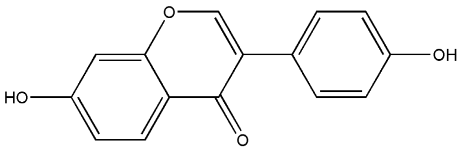

260

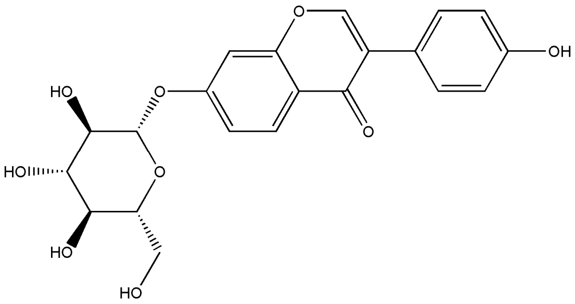

261

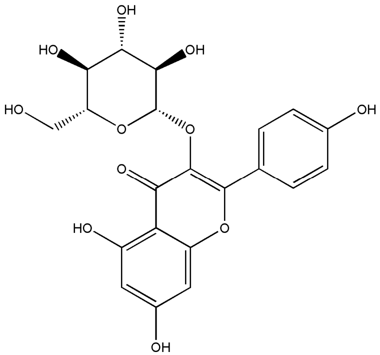

262

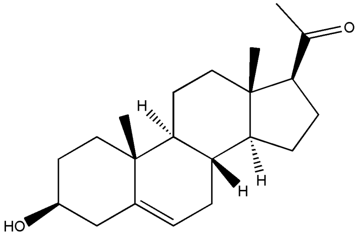

263

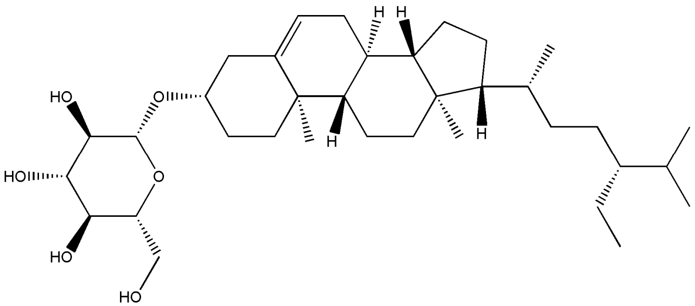

264

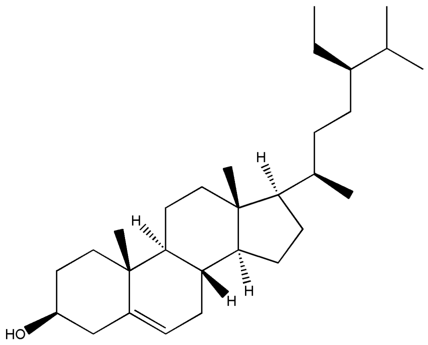

265

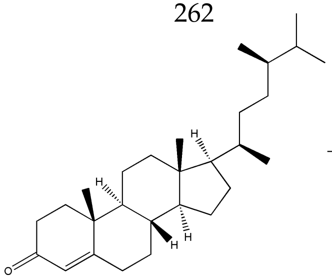

266

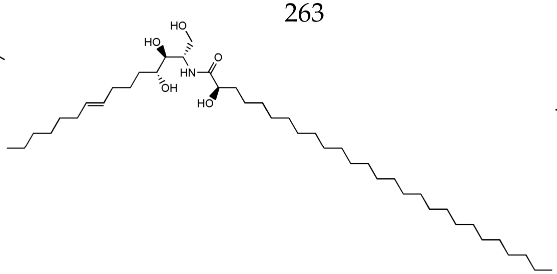

267

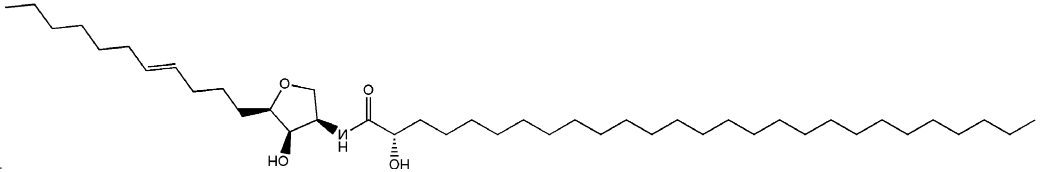

268

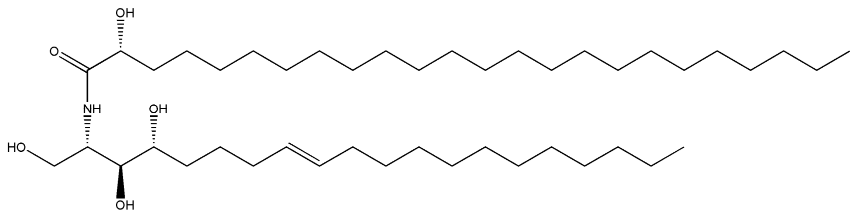

269

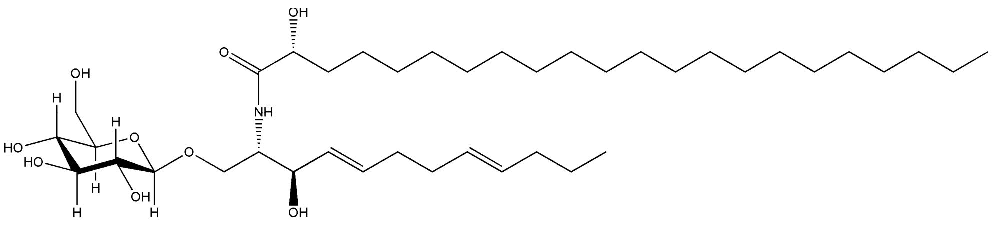

270

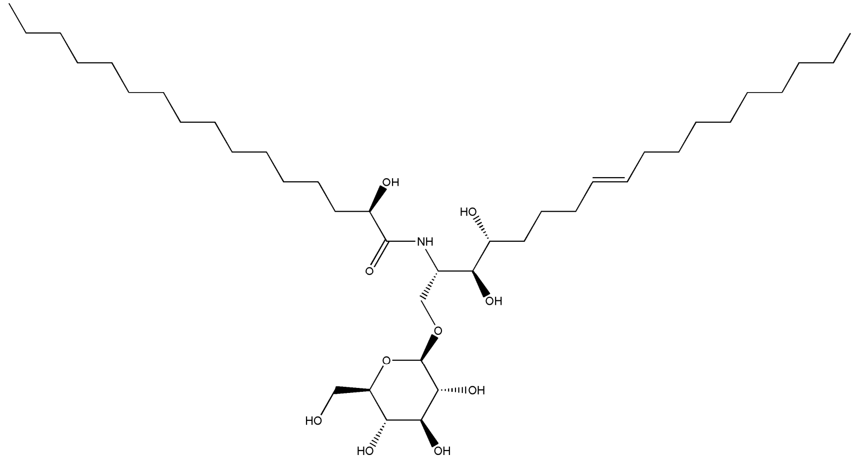

271

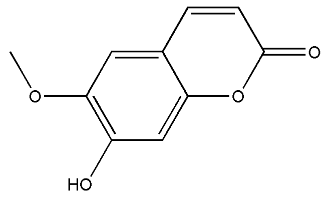

272

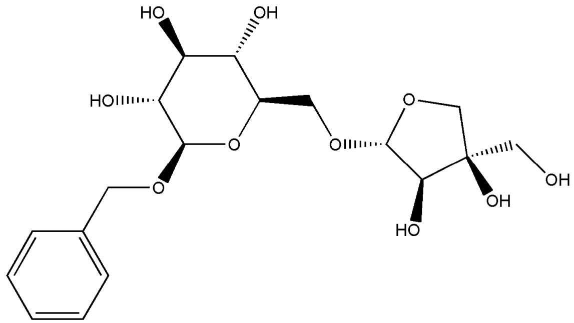

273

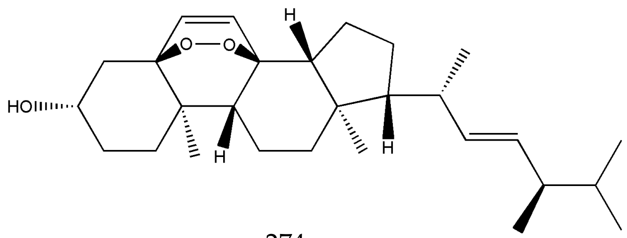

274

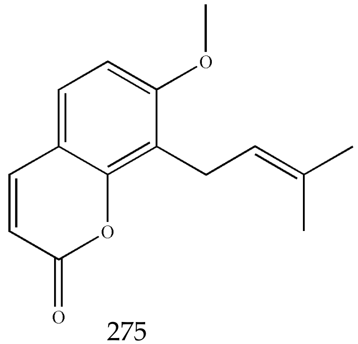

275

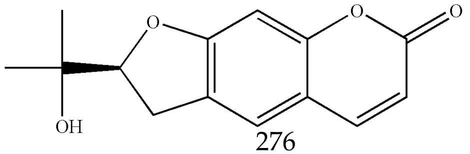

276

Figure S1. Chemistry composition of Szechwan Lovage Rhizome

Table S1. Chemical composition of Szechwan Lovage Rhizome

No.	Compound name	Ref.
1	Z-ligustilide	[1]
2	Senkyunolide A	[2]
3	Z-butylidenephthalide	[3]
4	E-ligustilide	[1]
5	Butylphthalide	[4]
6	Cnidilide	[5]
7	Neocnidilide	[5]
8	Senkyunolide H	[6]
9	Senkyunolide I	[7]
10	Senkyunolide J	[6]
11	Z-6, 7-epoxyligustilide	[8]
12	3-Carboxyrthyl - Phthalide	[9]
13	Senkyunolide B	[10]
14	Senkyunolide C	[11]
15	Senkyunolide D	[12]
16	Z-senkyunolide E	[13]
17	E-senkyunolide E	[7]
18	Senkyunolide F	[7]
19	Senkyunolide G	[6]
20	Senkyunolide K	[14]
21	Senkyunolide L	[14]
22	Senkyunolide M	[14]
23	Senkyunolide N	[7]
24	Senkyunolide Q	[15]
25	Senkyanolide R	[7]
26	Senkyunolide S	[7]
27	Chuanxiongnolide R1	[16]
28	Chuanxiangnolide R2	[16]
29	4-Hydroxyl-3-butylphthalide	[17]
30	4, 7-Dihydroxy-3-butylphthalide	[18]
31	Z-4, 5-Dihydroxyl-3-butylidenephthalide	[11]
32	2-(1-oxopentyl)-Benzoic acid methyl ester	[13]
33	Ligusticoside A	[19]
34	(+)-Chuanxiongin A	[20]
35	(-)-Chuanxiongin A	[20]
36	Chuanxiongin B	[20]
37	Chuanxiongin C	[20]
38	Chuanxiongin D	[20]
39	Chuanxiongin E	[20]
40	Chuanxiongin F	[20]
41	Ligusticumalide A	[21]
42	Ligusticumolide B	[21]
43	Ligusficumolide C	[21]
44	Ligusticumolide D	[21]
45	Ligusticumolide E	[21]
46	Ligusticumolide F	[21]
47	Ligusticumolide G	[21]
48	10R-1(3H)-Isobenzofuranone	[21]

49	10S-1(3H)-Isobenzofuranone	[21]
50	(+)-Neophathalide A	[22]
51	(-)-Neophathalide A	[22]
52	(+)-Neophathalide B	[22]
53	(-)-Neophathalide B	[22]
54	Ligubenzocycloheptanone A	[23]
55	(+)-4, 11-Dihydroxy-2-dodecene-5-lactose	[24]
56	Thiosenkyunolide C	[25]
57	6-Hydroxy-7-(2-carboxyl-2-hydroxyethylthio)-3-(2-hydroxy-butylidene)-4, 5, 6, 7-tetrahydrophthalide	[26]
58	Chuanxiongoside A	[27]
59	Chuanxiongoside B	[27]
60	Ligusticoside B	[27]
61	Celephthalide A	[27]
62	Icariside F2	[27]
63	(3Z, 3aZ)-3-Butylidene-b, 7-dihydroxy-4, 5, 6,7-tetrahydro-phthalide-7-O-α-D-glucopyranosyl- (1→2)-β-D-fructo-furanoside	[26]
64	3-(3 ,β-D-glucapyranosyloxy-butylidene)- 7-hydroxy-phthalide	[26]
65	Thiosenkyunolide A	[28]
66	Thiosenkyunolide B	[28]
67	Ligusticumside A	[28]
68	Ligusticumside B	[28]
69	Ligusticumside C	[28]
70	Ligusticumside D	[28]
71	Ligusticumside E	[28]
72	Ligusticumside F	[28]
73	Ligusticumside G	[28]
74	Ligustieumside H	[28]
75	3-Butylidene-6-hydroxy-7-ethoxy-phthalide	[29]
76	3-Eugenolsenkyunolide	[30]
77	4-Angeloylsenkyunolide K	[30]
78	Chuanxiongnolide L1	[31]
79	Ligusticumalides A	[32]
80	Ligusticumalides B	[32]
81	Falonolide A	[30]
82	Falonolide B	[30]
83	Senkyunolide O	[33]
84	Senkyunolide P	[33]
85	(+)-Levistolide A	[34]
86	(-)-Levistolide A	[35]
87	3,8-Dihydro-diligustilide	[33]
88	Tokinolide B	[33]
89	Ansaspirolide	[33]

90	(+)-Riligustilide	[34]
91	(-)-Riligustilide	[34]
92	Angelicide	[36]
93	Z,Z'-3,3',8,8'-Diligustilide	[33]
94	Z-Ligustilide dimer E-232	[37]
95	Chuanxiongnolide A	[33]
96	Chuanxiongnolide B	[33]
97	Wallichilide.	[33]
98	Chuanxiongdiolide R1	[10]
99	Chuanxiongdiolide R2	[16]
100	Chuanxiongdiolide R3	[38]
101	Chuanxiongdiolides R4	[39]
102	Chuanxiongdiolides R5	[39]
103	Chuanxiongdiolides R6	[39]
104	4,5-Dehydro-diligustilide	[40]
105	Chuanxiongnolide E	[41]
106	Z,Z'-3,3'a,7,7'a-Diligustilide	[42]
107	(+)-Demethywallichilide	[43]
108	(-)-Demethywallichilide	[43]
109	3,8-Dihydro-levistilide A	[42]
110	Angelicolide	[44]
111	Chuanxiongdiolide A	[45]
112	Chuanxiongdiolide B	[45]
113	(3Z)-(3aR,6S,3'R,8S)-3a,8',6,3'-Diligustilide	[42]
114	Tokinolide A	[46]
115	Chaxiongnolide D	[42]
116	Neodiligustilide	[47]
117	(+)-Chuanxiongdiolide R7	[34]
118	(-)-Chuanxiongdiolide R7	[34]
119	(+)-Chaxiongnolide G	[34]
120	(-)-Chaxiongnolide G	[41]
121	(+)-Z-3',8',3'a,7'a-Tetrahydro-6,3',7,7'a-diligustilide-8'- one	[48]
122	(-)-Z-3',8',3'a,7'a-Tetrahydro-6,3',7,7'a-diligustilide-9'-one	[34]
123	(+)-Gelispirolide	[34]
124	(-)-Gelispirolide	[49]
125	(3′Z)-(3S,8S,3a′S,6′R)-4,5- Dehydro-3.3a′,8.6′-diligustilide	[38]
126	(+)-Spiroligustolides A	[50]
127	(-)-Spiroligustolides A	[50]
128	(+)-Spiroligustolides B	[50]
129	(-)-Spiroligustolides B	[50]
130	1'', 3'-Seco-1'-ethoxy-3, 3a', 8, 6'-diligustilide.	[29]
131	Tokiaerialide	[39]
132	Methyl ester derived from angeolide	[39]
133	3Z,3'E-6.6‘,7.3'a-Diligustilide	[51]

134	3,3'Z-6.7',7.6-Diligustilide	[51]
135	3Z-6S,7R,3'S,8'R-6.8',7.3'-Diligustilide	[51]
136	(+)-6-3'a,7-6'-Isowallichilide	[52]
137	(-)-6-3'a,7-7'-Isowallichilide	[52]
138	Levistolide B	[53]
139	Chuanxiongdiolide R8	[30]
140	Chuanxiongdiolide R9	[30]
141	Chuanxiongdiolide R10	[30]
142	Chuanxiongnolide L2	[31]
143	Chuanxiongaolide L3	[31]
144	Chuanxiongnolide L4	[31]
145	Chuanxiongnolide L5	[31]
146	Chuanliguspirolide	[54]
147	Nicotinic acid	[55]
148	Cinnamic acid	[56]
149	Salicylic acid	[57]
150	Phthalic acid	[58]
151	*p*-Hydroxybenzoic acid	[59]
152	Ferulic acid	[60]
153	Caffeic acid	[61]
154	Protocatechuic acid	[61]
155	2-Methoxy-4-(3-methoxy-1-propenyl)-phenol	[13]
156	Vanillic acid	[62]
157	Vanillin	[63]
158	Gallic acid	[59]
159	Sinapic acid	[64]
160	Chrysophanol	[13]
161	Chlorogenic acid	[65]
162	Succinic acid	[66]
163	Palmitic acid	[67]
164	Linoleic acid	[68]
165	Oeic acid	[68]
166	Folic acid	[55]
167	Folinic acid	[55]
168	Augustic acid	[69]
169	Ligusticumacid A	[70]
170	Ligusticumacid B	[70]
171	Ligusticumacid C	[70]
172	Ligusticumaldehyde A	[70]
173	Ligusticumacid D	[70]
174	Ligusticumacid E	[70]
175	Ligusticumacid F	[70]
176	(*S*)-2-(2-carboxyl-2-hydroxyethylthio)-Ferulic acid	[71]
177	(*E*)-2-Methoxy-4-(3-(methylsulfonyl) prop-1-en-1-yl) phenol	[71]

178	3-Hydroxy-4-methoxypropiophenone-3-O-β-D-apiofuranosyl-(1--6)-β-D-glucopyranoside	[71]
179	β-D-Apiofuranosyl-(1--6)β-D-glucopyranosyl-3, 4-dimethoxy-benzoate	[71]
180	2, 3-Bis(4-hydroxy-3-methoxyphenyl) cyclopent-2-enone	[72]
181	4, 5-di-O-Caffeoyldaucic acid methyl ester	[72]
182	(+)-Ligusticumtone	[72]
183	(-)-Ligusticumtone	[72]
184	Lignoceric acid	[73]
185	Ethyl ferulate	[74]
186	Neochlorogenic acid	[75]
187	Tetradecanoic acid	[75]
188	(*E*)-Coniferyl alcohol	[76]
189	Sedanonic acid	[77]
190	Coniferyl ferulate	[78]
191	4-[(*E*)-3- ethoxy -1- propenyl] -2- methoxyphenol	[79]
192	(+)-ligustchuanes A	[80]
193	(-)-ligustchuanes A	[80]
194	(+)-ligustchuanes B	[80]
195	(-)-ligustchuanes B	[80]
196	Ligusticumin A	[32]
197	Ligusticumin B	[32]
198	Ligusticumin C	[32]
199	3,4- Dimethoxy -5- hydroxyphenylacetone	[79]
200	4- Hydroxy -3- methoxyphenylacetone	[79]
201	3,4- Dimethoxyphenylacetone	[79]
202	Tetramethylpyrazine	[81]
203	L-Isobutyl-L-valine anhydride	[81]
204	L-Isoleucinc-L-valine anhydride	[81]
205	L-Valine-L-valine anhydride	[81]
206	Perlolyrine	[81]
207	1-β-Ethylacrylate-7-aldehydo-β-carboline	[33]
208	1-Acetyl -β-carboline	[33]
209	Adenosine	[33]
210	Uracil	[33]
211	Adenine	[33]
212	Trimethylamine	[33]
213	Choline	[33]
214	Aurantiamide	[82]
215	Inosine	[82]
216	Uridine	[82]
217	Liguadenosine A	[83]
218	Liguadenosine B	[83]
219	2′-O-Methyladenosine	[83]

220	2′-O-Methyluridine	[83]
221	5-Methyluridine	[83]
222	Vernine	[76]
223	LCP-I-I	[84]
224	LCP-II-I	[85]
225	LCP70-2A	[86]
226	LCP	[87]
227	LCPXP-1a	[88]
228	LCPXP-3a	[88]
229	LCX0	[89]
230	LCX1	[89]
231	LCX2	[89]
232	Xiongterpene	[69]
233	Aromadendrane-4β,10β-diol	[19]
234	Aromadendrane-4α, 10β-diol	[19]
235	Aromadendrane-4α, 10α-diol	[19]
236	(-)-Alloaromadendrane-4β, 10α, 13, 15-tetrol	[9]
237	4-Pentylcyclo-hex-3-ene- 1α-2β-diol	[73]
238	4S-*p*-Menth-1-ene-4, 7diol	[19]
239	(-)-β-Elemene	[75]
240	Squalene	[75]
241	α-Pinene	[75]
242	Terpinen-4-ol	[75]
243	Spathulenol	[75]
244	Ethyl ferulate	[75]
245	Terpinolene	[75]
246	3-Carene	[75]
247	β-Eudesmo	[75]
248	Eudesma-4,11-dlene	[90]
249	6-Butyl-1,4-cycloheptadiene	[90]
250	Terpinene	[90]
251	(1S, 2R, 4R, 5S)-14-Deoxy-aspergiketone	[91]
252	Aspergiketone	[91]
253	AM6898B	[91]
254	Boydenes A	[91]
255	(20S)-Ginsenoside Rh1	[92]
256	(20R)-Ginsenoside Rh1	[92]
257	(20R)-Ginsenoside Rg3	[92]
258	Apigenin	[90]
259	Quercetin	[82]
260	Daidzein	[82]
261	Daidzin	[82]
262	Kaempferol-3 — O-β-D-glucopyranoside （=astragalin）	[82]
263	Pregnenolone	[81]

264	Daucosterol	[67]
265	β-Sitosterol	[9]
266	Campest-4-en-3-one	[9]
267	(2R)-2-Hydroxy-N [(2S, 3S, 4R, 8E)-1，3, 4-trihydroxypentadec-8-en-2-yl heptacosanamide	[93]
268	(2R)-2-Hydroxy-N- {(3S, 4S, 5S)-4-hydroxy-5-[(4E)-undec-4-En-1-yl tetrahydrofuran-3-yl} heptacosanamide	[93]
269	(2R) 2-Hydroxy-N- [(2S, 3S, 4R, 8E)-1,3, 4-trihydroxyicos-8-en-2-yl tetracosanamide	[93]
270	(2R)-N-[(2S, 3R, 4E, 8E)-1-(β-D-glucopyranosyloxy)-3-hydroxydodeca-4,8-dien-2-] yt-2-Hydroxydo-cosanamide	[93]
271	(2R)-N- [(2S, 3S, 4R, 8E)-1-(β-D-glucopyranosyloxy)-3,4-dihydroxyoctadee-8-en-2-] yl-2-Hydroxyhexadecanamide	[93]
272	Scopoletin	[73]
273	Icariside F2	[73]
274	Ergosterol peroxide	[73]
275	Osthole	[75]
276	Marmesin	[75]

References

1. Zhong, F.; Yang, L.; Ji, L.; Hu, S.; Fu, G. Studies on the Essential Oils in Ligusticum chuanxiong Hort. of Different Habitats and Species. *China J. Chin. Mater. Med.* **1996**, *03*, 147-151.
2. Yamagishi, T.; Kaneshima, H. Constituents of Cnidium officinale Makino. Structure of senkyunolide and gas chromatography-mass spectrometory of the related phthalides. *Yakugaku zasshi : Journal of the Pharmaceutical Society of Japan* **1977**, *97*, 237-243, doi:10.1248/yakushi1947.97.3_237.
3. Gijbels, M.J.; Scheffer, J.J.; Baerheim Svendsen, A. Phthalides in the essential oil from roots of Levisticum officinale. *Planta Med.* **1982**, *44*, 207-211, doi:10.1055/s-2007-971448.
4. Bohrmann, H.; Stahl, E.; Mitsuhashi, H. Studies of the constituents of umbelliferae plants. 8. Chromatographic studies on the constituents of Cnidium officinale Makino. *Chem. Pharm. Bull.* **1967**, *15*, 1606-1608, doi:10.1248/cpb.15.1606.
5. Bohrmann, H.; Stahl, E.; Mitsuhashi, H. Studies of the Constituents of Umbelliferae Plants. XIII. Chromatographic Studies on the Constituents of Cnidium officinale MAKINO. *Chem. Pharm. Bull.* **1967**, *15*, 1606-1608, doi:10.1248/cpb.15.1606.
6. Kobayashi, M.; Fujita, M.; Mitsuhashi, H. Studies on the Constituents of Umbelliferae Plants. XV. Constituents of Cnidium officinale: Occurrence of Pregnenolone, Coniferylferulate and Hydroxyphthalides. *Chem. Pharm. Bull.* **1987**, *35*, 1427-1433, doi:10.1248/cpb.35.1427.
7. Takashi, N.; Takao, K.; Kazuaki, N.; Yukinobu, I.; Minoru, O.; Hiroshi, M. Two phthalides from Ligusticum chuangxiong. *Phytochemistry* **1992**, *31*, 639-642, doi:https://doi.org/10.1016/0031-9422(92)90051-Q.
8. Zhang, Q.; Wang, M.; Wang, Q.; Zhao, H.; Zhang, Z.; Yu, H.; Liu, Y.; Fu, S.; Lu, Z.; Huang, Z.; et al. Characterization of the potential new phthalides in Ligusticum chuanxiong Hort. using ultra-

performance liquid chromatography coupled with quadrupole time of flight tandem mass spectrometry. *J. Sep. Sci.* **2017**, *40*, 2123-2130, doi:10.1002/jssc.201601443.
9. Miao, C.; Wu, S.; Luo, B.; Wang, J.; Chen, Y. A new sesquiterpenoid from Ligusticum chuanxiong Hort. *Fitoterapia* **2010**, *81*, 1088-1090, doi:10.1016/j.fitote.2010.07.001.
10. Pushan, W.; Xuanliang, G.; Yixiong, W.; Fukuyama, Y.; Miura, I.; Sugawara, M. Phthalides from the rhizome of Ligusticum wallichii. *Phytochemistry* **1984**, *23*, 2033-2038, doi:https://doi.org/10.1016/S0031-9422(00)84965-1.
11. Kaouadji, M.; Puech-Baronnat, M.; Mariotte, A.-M. (Z) Ligustilidiol, nouveau phtalide hydroxyle isole de Ligusticum wallichii Franch. *Tetrahedron Letters* **1983**, *24*, 4675-4676, doi:https://doi.org/10.1016/S0040-4039(00)86224-3.
12. Wang, Z.; Li, S.; Yan, F.; Li, Y. Synthesis of (Z)-3-(2-Hydroxybutylidene)phthalide. *Synthetic Communications* **1994**, *24*, 3135-3139, doi:10.1080/00397919408011328.
13. Naito, T.; Niitsu, K.; Ikeya, Y.; Okada, M.; Mitsuhashi, H. A phthalide and 2-farnesyl-6-methyl benzoquinone from Ligusticum chuangxiong. *Phytochemistry* **1992**, *31*, 1787-1789, doi:https://doi.org/10.1016/0031-9422(92)83148-R.
14. Huang, J.; Lu, X.Q.; Zhang, C.; Lu, J.; Li, G.Y.; Lin, R.C.; Wang, J.H. Anti-inflammatory ligustilides from Ligusticum chuanxiong Hort. *Fitoterapia* **2013**, *91*, 21-27, doi:10.1016/j.fitote.2013.08.013.
15. Kobayashi, M.; Mitsuhashi, H. Studies on the Constituents of Umbelliferae Plants. XVII. Structures of Three New Ligustilide Derivatives from Ligusticum wallichii. *Chem. Pharm. Bull.* **1987**, *35*, 4789-4792, doi:10.1248/cpb.35.4789.
16. Kobayashi, M.; Fujita, M.; Mitsuhashi, H. Components of Cnidium officinale Makino: occurrence of pregnenolone, coniferyl ferulate, and hydroxyphthalides. *Chem. Pharm. Bull.* **1984**, *32*, 3770-3773, doi:10.1248/cpb.32.3770.
17. Zhang, B.; Sun, J.M.; Chang, R.L.; Zhang, H. Studies on the chemical constituents of the root and rhizoma of Ligusticum jeholense. *J. Chin. Med. Mater.* **2009**, *32*, 710-712.
18. Chang, X.L.; Ma, Y.B.; Zhang, X.M.; Jiang, Z.Y.; Chen, J.J. Studies on chemical constituents of rhizomes of Ligusticum chuanxiong. *China J. Chin. Mater. Med.* **2007**, *32*, 1533-1536.
19. Chang, X.L.; Jiang, Z.Y.; Ma, Y.B.; Zhang, X.M.; Tsim, K.W.; Chen, J.J. Two new compounds from the roots of Ligusticum chuanxiong. *J. Asian. Nat. Prod. Res.* **2009**, *11*, 805-810, doi:10.1080/10286020903071068.
20. Yang, J.; Feng, X.L.; Yu, Y.; Wang, Q.; Zou, J.; Wang, C.X.; Mu, Z.Q.; Yao, X.S.; Gao, H. Novel phthalide derivatives identified from Ligusticum chuanxiong (Chuanxiong). *Chin. Med.* **2016**, *11*, 10, doi:10.1186/s13020-016-0080-2.
21. Zhang, X.; Feng, Z.M.; Yang, Y.N.; Jiang, J.S.; Zhang, P.C. Bioactive butylphthalide derivatives from Ligusticum chuanxiong. *Bioorg. Chem.* **2019**, *84*, 505-510, doi:10.1016/j.bioorg.2018.12.032.
22. Zhang, X.; Yan, H.W.; Feng, Z.M.; Yang, Y.N.; Jiang, J.S.; Zhang, P.C. Neophathalides A and B, two pairs of unusual phthalide analog enantiomers from Ligusticum chuanxiong. *Org. Biomol. Chem.* **2020**, *18*, 5453-5457, doi:10.1039/d0ob01014f.
23. Han, B.; Zhang, X.; Feng, Z.M.; Jiang, J.S.; Li, L.; Yang, Y.N.; Zhang, P.C. Ligubenzocycloheptanone A, a Novel Tricyclic Butenolide with a 6/7/5 Skeleton from Ligusticum chuanxiong. *Sci. Rep.* **2016**, *6*, 28783, doi:10.1038/srep28783.
24. Li, X.-R.; Ma, C.; Guo, L.; Lu, J.; Xiong, L.; Zhou, Q.-M. A new lactone from Alternaria sp. lS275, an endophytic fungus of Ligusticum chuanxiong. *Chin. Tradit. Herb. Drugs* **2019**, *50*, 4859-4862.

25. Zhang, X.; Han, B.; Feng, Z.; Jiang, J.; Yang, Y.; Zhang, P. Bioactive thionic compounds and aromatic glycosides from Ligusticum chuanxiong. *Acta. Pharm. Sin. B* **2018**, *8*, 818-824, doi:10.1016/j.apsb.2018.04.002.
26. Yuan, X.; Han, B.; Feng, Z.M.; Jiang, J.S.; Yang, Y.N.; Zhang, P.C. Three butylphthalide derivatives from the Rhizome of Ligusticum chuanxiong. *Acta. Pharm. Sin. B* **2020**, *55*, 2674-2678, doi:10.16438/J.0513-4870.2020-0887.
27. Li, L.-J.; Su, Y.-F.; Yan, S.-L. Three new phthalide glycosides from the rhizomes of Ligusticum chuanxiong. *Phytochemistry Letters* **2016**, *17*, 14-17, doi:https://doi.org/10.1016/j.phytol.2016.05.013.
28. Zhang, X.; Han, B.; Feng, Z.-M.; Yang, Y.-N.; Jiang, J.-S.; Zhang, P.-C. Phthalide derivatives from Ligusticum chuanxiong. *RSC Advances* **2017**, *7*, 37478-37486, doi:10.1039/C7RA06813A.
29. Zhang, K. Chemical Constituents from the Rhizome of Ligusticum chuanxiong Hort. and Their Nrf2 Inducing Activity. Master, Shandong University, Shandong, 2021.
30. Tan, Y.-Z.; Yan, H.-L.; Liu, Y.-Y.; Yan, Y.-M.; Wang, L.; Qiao, J.-X.; Wu, J.; Tian, Y.; Peng, C. Structurally diverse phthalides from fibrous roots of Ligusticum chuanxiong Hort. and their biological activities. *Fitoterapia* **2024**, *175*, 105882, doi:https://doi.org/10.1016/j.fitote.2024.105882.
31. Wei, W.; Wu, X.-W.; Yang, X.-W. Novel phthalide derivatives from the rhizomes of Ligusticum chuanxiong and their inhibitory effect against lipopolysaccharide-induced nitric oxide production in RAW 264.7 macrophage cells. *RSC Advances* **2016**, *6*, 61037-61046, doi:10.1039/C6RA10023F.
32. Yang, M.-L.; Yang, H.-D.; Tang, Z.-S.; Hu, X.-H.; Zhou, R.; Xue, T.-T.; Ma, K.; Ji, C.; Xu, H.-B. Lignan and Phthalide Derivatives from the Rhizome of Ligusticum chuanxiong (Rhizoma chuanxiong) and Evaluation of Their anti-Xanthine Oxidase Activities. *ACS Omega* **2023**, *8*, 39855-39864, doi:10.1021/acsomega.3c06172.
33. Li, W.; Tang, Y.; Chen, Y.; Duan, J.A. Advances in the chemical analysis and biological activities of chuanxiong. *Molecules (Basel, Switzerland)* **2012**, *17*, 10614-10651, doi:10.3390/molecules170910614.
34. Huang, L.; Peng, C.; Guo, L.; Feng, R.; Shu, H.Z.; Tian, Y.C.; Zhou, Q.M.; Xiong, L. Six pairs of enantiomeric phthalide dimers from the rhizomes of Ligusticum chuanxiong and their absolute configurations and anti-inflammatory activities. *Bioorg. Chem.* **2022**, *127*, 105970, doi:10.1016/j.bioorg.2022.105970.
35. Lee, T.F.; Lin, Y.L.; Huang, Y.T. Studies on antiproliferative effects of phthalides from Ligusticum chuanxiong in hepatic stellate cells. *Planta Med.* **2007**, *73*, 527-534, doi:10.1055/s-2007-981520.
36. Ang, M. Analysis of the Ingredients of Angelica sinensis - Determination of the Structure of Angelicide. 1984.
37. Hon, P.-M.; Lee, C.-M.; Choang, T.F.; Chui, K.-Y.; Wong, H.N.C. A ligustilide dimer from angelica sinensis. *Phytochemistry* **1990**, *29*, 1189-1191, doi:https://doi.org/10.1016/0031-9422(90)85426-G.
38. Wei, W.; Xu, W.; Yang, X.W. Two new phthalide dimers from the rhizomes of Ligusticum chuanxiong. *J. Asian. Nat. Prod. Res.* **2017**, *19*, 704-711, doi:10.1080/10286020.2016.1275584.
39. Tang, F.; Yan, Y.M.; Yan, H.L.; Wang, L.X.; Hu, C.J.; Wang, H.L.; Ao, H.; Peng, C.; Tan, Y.Z. Chuanxiongdiolides R4 and R5, phthalide dimers with a complex polycyclic skeleton from the

aerial parts of Ligusticum chuanxiong and their vasodilator activity. *Bioorg. Chem.* **2021**, *107*, 104523, doi:10.1016/j.bioorg.2020.104523.

40. Lim, L.S.; Shen, P.; Gong, Y.H.; Yong, E.L. Dimeric progestins from rhizomes of Ligusticum chuanxiong. *Phytochemistry* **2006**, *67*, 728-734, doi:10.1016/j.phytochem.2006.01.024.
41. Wei, Q.; Yang, J.; Li, L.; Su, Y.; Wang, A. Novel phthalide dimers from the aerial parts of Ligusticum sinense Oliv cv. Chaxiong. *Fitoterapia* **2019**, *137*, 104174, doi:10.1016/j.fitote.2019.104174.
42. Yan, H.; Zhou, Y.; Tang, F.; Wang, C.; Wu, J.; Hu, C.; Xie, X.; Peng, C.; Tan, Y. A comprehensive investigation on the chemical diversity and efficacy of different parts of Ligusticum chuanxiong. *Food Funct.* **2022**, *13*, 1092-1107, doi:10.1039/D1FO02811A.
43. León, A.; Delgado, G. Diligustilide: Enantiomeric Derivatives, Absolute Configuration and Cytotoxic Properties. *J. Mex. Chem. Soc.* **2012**, *56*, 222-226, doi:10.29356/jmcs.v56i2.326.
44. Banerjee, S.K.; Gupta, B.D.; Sheldrick, W.S.; Höfle, G. Lactonic Constituents of Angelica glauca. *Liebigs Ann. Chem.* **2006**, *1984*, 888-893, doi:10.1002/jlac.198419840506.
45. Huang, J.; Lu, X.Q.; Lu, J.; Li, G.Y.; Wang, H.Y.; Li, L.H.; Lin, R.C.; Wang, J.H. Two new phthalides with BuChE inhibitory activity from Ligusticum chuanxiong. *J. Asian Nat. Prod. Res.* **2013**, *15*, 1237-1242, doi:10.1080/10286020.2013.825610.
46. Gong, W.; Zhou, Y.; Li, X.; Gao, X.; Tian, J.; Qin, X.; Du, G. Neuroprotective and Cytotoxic Phthalides from Angelicae Sinensis Radix. *Molecules (Basel, Switzerland)* **2016**, *21*, doi:10.3390/molecules21050549.
47. Chen, Q.C.; Lee, J.; Jin, W.; Youn, U.; Kim, H.; Lee, I.S.; Zhang, X.; Song, K.; Seong, Y.; Bae, K. Cytotoxic constituents from angelicae sinensis radix. *Arch. Pharm. Res.* **2007**, *30*, 565-569, doi:10.1007/bf02977650.
48. Su, D.M.; Yu, S.S.; Qin, H.L. New dimeric phthalide derivative from Angelica sinensis. *Acta. pharm. Sin.* **2005**, *40*, 141-144.
49. Zou, J.; Chen, G.D.; Zhao, H.; Wang, X.X.; Zhang, Z.J.; Qu, Y.B.; He, R.R.; So, K.F.; Yao, X.S.; Gao, H. Triangeliphthalides A-D: bioactive phthalide trimers with new skeletons from Angelica sinensis and their production mechanism. *Chem. Commun.* **2019**, *55*, 6221-6224, doi:10.1039/c9cc02681a.
50. Wang, L.Y.; Li, W.Y.; Zhou, H.F.; Zhao, X.Y.; Li, X.N.; Wu, X.D.; Zhao, Q.S. Spiroligustolides A and B: Two pairs of enantiomeric spiro-orthoester-containing phthalide dimers as Ca(v)3.1 calcium channel inhibitors from Ligusticum Chuanxiong Hort. *Bioorg. Chem.* **2022**, *123*, 105749, doi:10.1016/j.bioorg.2022.105749.
51. Zhang, L.-Y. Isolation of active phthalides regulating nuclearreceptor TR3 from Angelicae Sinensis Radix, ChuanxiongRhizoma and Ligustici Rhizoma et Radix. Master, Xiamen University, Xiamen, 2020.
52. Fang, X.; Ma, Q.; Feng, Y.; Liang, S. (±)-6-3'a,7-6'-Isowallichilide:A pair of enantiomeric phthalide dimers from Ligusticum chuanxiong with new 6-3'a,7-6' dimerization sites. *J. Chinese Chemical Letters* **2020**, *31*, 1251-1253.
53. Cichy, M.; Wray, V.; Höfle, G. Neue Inhaltsstoffe von Levisticum officinale Koch (Liebstöckel). *Eur. J. Org. Chem.* **1984**, *1984*, 397-400, doi:https://doi.org/10.1002/jlac.198419840221.
54. Zheng, Y.R.; Wei, W.; Yang, X.W. Chuanliguspirolide, a new butylphthalide derivative from Chuanxiong Rhizoma and its inhibition on NO production in LPS-activated RAW264.7 and BV2 cell lines. *Chin. Tradit. Herb. Drugs* **2018**, *49*, 1497-1503, doi:10.7501/j.issn.0253-2670.2018.07.002.

55. Huang, W.Y.; Sheu, S.J. Separation and identification of the organic acids in Angelicae Radix and Ligustici Rhizoma by HPLC and CE. *J. Sep. Sci.* **2006**, *29*, 2616-2624, doi:10.1002/jssc.200600136.
56. Zou, J.; Gao, P.; Hao, X.; Xu, H.; Zhan, P.; Liu, X. Recent progress in the structural modification and pharmacological activities of ligustrazine derivatives. *Eur. J. Med. Chem.* **2018**, *147*, 150-162, doi:10.1016/j.ejmech.2018.01.097.
57. Chen, X.; Mao, J.; Wen, F.; Xu, X. Determination of Phenolic Acids in Botanical Pharmaceutical Products by Capillary Electrophoresis with Chemiluminescence Detection. *Analytical Letters* **2021**, *54*, 817-829, doi:10.1080/00032719.2020.1783675.
58. Xu, F.; Zhang, L.; Zhao, X.; Zhou, Q.-L.; Liu, G.-X.; Yang, X.-W.; Yang, D.-H.; Cai, S.-Q. Eleven Absorbed Constituents and 91 Metabolites of Chuanxiong Rhizoma Decoction in Rats. *World J. Tradit. Chin. Med.* **2021**, *7*.
59. Chu, Q.C.; Zhang, D.L.; Zhang, H.T.; Ye, J.N. Study on Rhizoma Chuanxiong based on capillary electrophoresis with amperometric detection. *Chin. Chem. Lett.* **2010**, *21*, 217-220, doi:https://doi.org/10.1016/j.cclet.2009.10.009.
60. Sun, Y.; Wang, W. Ultrasonic extraction of ferulic acid from Ligusticum chuanxiong. *J. Chin. Inst. Chem. Eng.* **2008**, *39*, 653-656, doi:https://doi.org/10.1016/j.jcice.2008.05.012.
61. Xiao, Y.Q.; Li, L.; You, X.L.; Taniguchi, M.; Baba, K. Studies on chemical constituents of the rhizomae of Ligusticum chuanxiong. *China J. Chin. Mater. Med.* **2002**, *27*, 519-522.
62. Zhang, Y.N.; Yue, X.F.; Zhang, Z.Q. Study on the interactions between four components in Ligusticum chuanxiong rhizome and acceptors on cardiac muscle membrane. *China J. Chin. Mater. Med.* **2004**, *29*, 660-662.
63. Li, S.L.; Chan, S.S.; Lin, G.; Ling, L.; Yan, R.; Chung, H.S.; Tam, Y.K. Simultaneous analysis of seventeen chemical ingredients of Ligusticum chuanxiong by on-line high performance liquid chromatography-diode array detector-mass spectrometry. *Planta Med.* **2003**, *69*, 445-451, doi:10.1055/s-2003-39709.
64. Pu, Z.-H.; Meng, C.-W.; Zhuo, Y.-X.; Peng, C.; Du, J.-C.; Xiong, L. Chemical constituents from Ligusticum chuanxiong. *J. Chin. Med. Mater.* **2016**, *39*, 2529-2531.
65. Zhao, Y.X.; Ding, M.Y.; Liu, D.L. Phenolic acids analysis in ligusticum chuanxiong using HPLC. *J. Chromatogr. Sci.* **2005**, *43*, 389-393, doi:10.1093/chromsci/43.8.389.
66. Hao, S.-J.; Zhang, Z.-X.; Tian, Y.; Ma, Y.-P.; Peng, Q.; Shen, S.; Zhang, L.; Jin, H. Study on Chemical Constituents of Ligusticum Chuanxiong Hort. *Mod. Tradit. Chin. Med.* **2010**, *12*, 22-25+38.
67. Wang, W.-X.; Gu, M.; Jiang, X.-G.; Gu, Z.-L.; Fan, P.-S. Studies on chemical constituents of Ligusticum chuanxiong. *Chin. Tradit. Herb. Drugs* **2001**, *33*, 6-7.
68. Wu, Q.; Yang, W.-X. GC-MS analysis of essential oil from rhizomes of Ligusticum chuanxiong cultivated in GAP Base for Chinese Medicinal Materials of China. *China J. Chin. Mater. Med.* **2008**, *33*, 276-280.
69. Chang, X.-L.; Ma, Y.-B.; Zhang, X.-M.; Zhi-Yong, J.; Chen, J.-J. Studies on chemical constituents of rhizomes of Ligusticum chuanxiong. *China J. Chin. Mater. Med.* **2007**, *32*, 1533-1536.
70. Zhang, X.; Han, B.; Feng, Z.M.; Yang, Y.N.; Jiang, J.S.; Zhang, P.C. Ferulic acid derivatives from Ligusticum chuanxiong. *Fitoterapia* **2018**, *125*, 147-154, doi:10.1016/j.fitote.2018.01.005.
71. Zhang, X.; Han, B.; Feng, Z.; Jiang, J.; Yang, Y.; Zhang, P. Bioactive thionic compounds and aromatic glycosides from Ligusticum chuanxiong. *Acta Pharm. Sin. B* **2018**, *8*, 818-824, doi:10.1016/j.apsb.2018.04.002.

72. Zhang, X.; Feng, Z.-M.; Yang, Y.-N.; Jiang, J.-S.; Zhang, P.-C. Phenolic acid derivatives from Ligusticum chuanxiong. *Phytochem. Lett.* **2019**, *33*, 114-118, doi:https://doi.org/10.1016/j.phytol.2019.08.010.
73. Han, W. Advances in Chemical Constituents and Pharmacological Effects of Ligusticum chuanxiong. *Mod. Chin. Med.* **2017**, *19*, 1341-1349.
74. Kong, L.; Yu, Z.; Bao, Y.; Su, X.; Zou, H.; Li, X. Screening and analysis of an antineoplastic compound in Rhizoma Chuanxiong by means of in vitro metabolism and HPLC-MS. *Anal. Bioanal. Chem.* **2006**, *386*, 264-274, doi:10.1007/s00216-006-0621-0.
75. Li, D.; Long, Y.; Yu, S.; Shi, A.; Wan, J.; Wen, J.; Li, X.; Liu, S.; Zhang, Y.; Li, N.; et al. Research Advances in Cardio-Cerebrovascular Diseases of Ligusticum chuanxiong Hort. *Front. Pharmacol.* **2021**, *12*, 832673, doi:10.3389/fphar.2021.832673.
76. Sun, J.-Y. I. Studies on the Chemical Constituents of Ligusticum chuanxiong Hort. and Codonopsis tangshen Oliv. II. Evaluation on Pharmacodynamics of Analgesia and Anti-inflammation of Anemone vitifolia Buch.-Ham. . Master, Nngxia University, Ningxia, 2017.
77. Beijing Institute of Pharmaceutical Industry. Chemical studies on the components of Ligusticum chuanxiong. *Chin. Pharm. J.* **1980**, *10*, 39.
78. Kobayashi, M.; Mitsuhashi, H. Studies on the Constituents of Umbelliferae Plants. XVII. Structures of Three New Ligustilide Derivatives from <I>Ligusticum wallichii</I>. *Chem. Pharm. Bull.* **1987**, *35*, 4789-4792, doi:10.1248/cpb.35.4789.
79. Liu, J.; Feng, M.-H.; Yang, X.-Q.; Li, Y.-T.; Jia, Z.-X.; Xiao, H.-B. Chemical Constituents of Ligusticum chuanxiong. *J. Chin. Med. Mater.* **2022**, *45*, 848-852.
80. Wan, S.J.; Ren, H.G.; Jiang, J.M.; Xu, G.; Xu, Y.; Chen, S.M.; Chen, G.; Zheng, D.; Yuan, M.; Zhang, H.; et al. Two Novel Phenylpropanoid Trimers From Ligusticum chuanxiong Hort With Inhibitory Activities on Alpha-Hemolysin Secreted by Staphylococcus aureus. *Front. Chem.* **2022**, *10*, 877469, doi:10.3389/fchem.2022.877469.
81. Yang, L.-H.; Xie, X.-Q.; Wang, L.; Wang, S.-B.; Zhan, K. Chemical Constituents from Ligusticum chuanxiong. *Lishizhen Med. Mater. Med. Res.* **2007**, *18*, 1576-1577.
82. Ren, D.-C.; Xie, N.; Yang, N.-Y.; Qian, S.-H.; Duan, J.A. Studies on the chemical constituents of the above-ground parts of Ligusticum chuanxiong. *China J. Chin. Mater. Med.* **2008**, *20*, 2417-2418.
83. Pu, Z.H.; Liu, J.; Peng, C.; Luo, M.; Zhou, Q.M.; Xie, X.F.; Chen, M.H.; Xiong, L. Nucleoside alkaloids with anti-platelet aggregation activity from the rhizomes of Ligusticum striatum. *Nat. Prod. Res.* **2019**, *33*, 1399-1405, doi:10.1080/14786419.2017.1416382.
84. Zou, Y.F.; Fu, Y.P.; Chen, X.F.; Austarheim, I.; Inngjerdingen, K.T.; Huang, C.; Eticha, L.D.; Song, X.; Li, L.; Feng, B.; et al. Purification and Partial Structural Characterization of a Complement Fixating Polysaccharide from Rhizomes of Ligusticum chuanxiong. *Molecules (Basel, Switzerland)* **2017**, *22*, doi:10.3390/molecules22020287.
85. Huang, C.; Cao, X.; Chen, X.; Fu, Y.; Zhu, Y.; Chen, Z.; Luo, Q.; Li, L.; Song, X.; Jia, R.; et al. A pectic polysaccharide from Ligusticum chuanxiong promotes intestine antioxidant defense in aged mice. *Carbohydr. Polym.* **2017**, *174*, 915-922, doi:10.1016/j.carbpol.2017.06.122.
86. Zhang, S.; An, L.; Li, Z.; Wang, X.; Wang, H.; Shi, L.; Bao, J.; Lan, X.; Zhang, E.; Lall, N.; et al. Structural elucidation of an immunological arabinan from the rhizomes of Ligusticum chuanxiong, a traditional Chinese medicine. *Int. J. Biol. Macromol.* **2021**, *170*, 42-52, doi:10.1016/j.ijbiomac.2020.12.069.

87. Zhang, W.-J.; Wang, P.; Lin, Q.; Li, Y.; Zhang, Q.-H.; Li, Y.-Q. Extraction processof three kinds of chinese herbs polysaccharides. *Chin. J. Exp. Tradit. Med. Formulae* **2011**, *17*, 19-23, doi:10.13422/j.cnki.syfjx.2011.16.018.
88. Zhong, C.; Liu, Z.; Zhang, X.; Pu, Y.; Yang, Z.; Bao, Y. Physicochemical properties of polysaccharides from Ligusticum chuanxiong and analysis of their anti-tumor potential through immunoregulation. *Food Funct.* **2021**, *12*, 1719-1731, doi:10.1039/d0fo02978e.
89. Hu, J.; Jia, X.; Fang, X.; Li, P.; He, C.; Chen, M. Ultrasonic extraction, antioxidant and anticancer activities of novel polysaccharides from Chuanxiong rhizome. *Int. J. Biol. Macromol.* **2016**, *85*, 277-284, doi:10.1016/j.ijbiomac.2015.12.046.
90. Shuai, S.Y.; Liu, S.S.; Liu, X.J.; Zhang, G.S.; Zheng, Q.; Yue, P.F.; Yang, M.; Hu, P.Y. Essential oil of Ligusticum chuanxiong Hort. Regulated P-gp protein and tight junction protein to change pharmacokinetic parameters of temozolomide in blood, brain and tumor. *J. Ethnopharmacol.* **2022**, *298*, 115646, doi:10.1016/j.jep.2022.115646.
91. Li, X.-H.; Hu, H.-W.; Tan, L.; Wu, W.-L.; Cao, Z.-X.; Gu, Y.-C.; Deng, Y.; Guo, D.-l. Sesquiterpenes Isolated from Aspergillus fumigatus, an Endophytic Fungus from Ligusticum wallichii. *Nat. Prod. Commun.* **2018**, *13*, 1934578X1801301101, doi:10.1177/1934578X1801301101.
92. Pu, Z.H.; Peng, C.; Su, H.G.; Meng, c.-w.; Xiong, L. Discovery of ginsenosides from Ligusticum chuanxiong and its significance. *Chin. Tradit. Herb. Drugs* **2017**, *48*, 3677-3681, doi:10.7501/j.issn.0253-2670.2017.18.002.
93. Yang, N.-Y.; Ren, D.-C.; Duan, J.-A.; Xu, X.-H.; Xie, N.; Tian, L.-J. Ceramides and Cerebrosides from Ligusticum chuanxiong Hort. *Helv. Chim. Acta* **2009**, *92*, 291-297, doi:https://doi.org/10.1002/hlca.200800240.
